# Ultrafast radiative heat transfer

**DOI:** 10.1038/s41467-016-0013-x

**Published:** 2017-02-23

**Authors:** Renwen Yu, Alejandro Manjavacas, F. Javier García de Abajo

**Affiliations:** 1 ICFO-Institut de Ciencies Fotoniques, The Barcelona Institute of Science and Technology, 08860 Castelldefels (Barcelona), Spain; 2 Department of Physics and Astronomy, University of New Mexico, 1919 Lomas Boulevard NE, Albuquerque, New Mexico 87131-0001 USA; 3 ICREA-Institució Catalana de Recerca i Estudis Avançats, Passeig Lluís Companys 23, 08010 Barcelona, Spain

## Abstract

Light absorption in conducting materials produces heating of their conduction electrons, followed by relaxation into phonons within picoseconds, and subsequent diffusion into the surrounding media over longer timescales. This conventional picture of optical heating is supplemented by radiative cooling, which typically takes place at an even lower pace, only becoming relevant for structures held in vacuum or under extreme thermal isolation. Here, we reveal an ultrafast radiative cooling regime between neighboring plasmon-supporting graphene nanostructures in which noncontact heat transfer becomes a dominant channel. We predict that more than 50% of the electronic heat energy deposited on a graphene disk can be transferred to a neighboring nanoisland within a femtosecond timescale. This phenomenon is facilitated by the combination of low electronic heat capacity and large plasmonic field concentration in doped graphene. Similar effects should occur in other van der Waals materials, thus opening an unexplored avenue toward efficient heat management.

## Abstract (web summary)

Electron relaxation, which is the dominant release channel of electronic heat in nanostructures, occurs with characteristic times of several picoseconds. Here, the authors predict that an ultrafast (femtosecond) radiative cooling regime takes place in plasmonically active neighboring graphene nanodisks prior to electron relaxation.

## Introduction

Optical, electrical, and mechanical dissipation in nanoscale devices produces heat accumulation that can result in structural damage and poor performance. Understandably, heat management constitutes an important aspect when designing thermoelectric^1^, optoelectronic^2^, electromechanical^3^, and photovoltaic^4^ elements, as well as recently proposed thermal analogs of electronic devices^5,6^. However, the relatively slow thermal conduction in most materials^7^ imposes a serious limitation. Finding new means of cooling nanostructures is, therefore, critical. An interesting possibility is provided by coupling to radiative degrees of freedom. Indeed, the absorption and emission of radiation by a material structure contributes to reach thermal equilibrium with other surrounding structures and the electromagnetic environment. This is the dominant cooling channel for thermally isolated structures^8^, in which energy is released through the emission of photons with wavelengths $\sim {\lambda }_{T}\mathrm{=2}\pi \hbar c/{k}_{{\rm{B}}}T$ (i.e., the thermal wavelength at temperature *T*). When the structures are separated by vacuum gaps of large size compared with *λ*_*T*_, the Planck and Kirchhoff laws determine the exchanged power^9^. In contrast, for neighboring objects separated by a small distance compared with *λ*_*T*_, radiative heat transfer is dominated by additional channels mediated by evanescent waves^10–12^. These can produce rates exceeding the black-body limit by several orders of magnitude, enhanced by near-field coupling of resonances supported by the nanostructures, thus emerging as a potentially relevant transfer mechanism in solid-state devices.

Following pioneering observations of near-field radiative energy transfer between two conducting plates^10,11^, a theoretical explanation was offered^12^ based on the effect of thermal fluctuations in the electrical current of the involved surfaces. Further experimental^13–22^ and theoretical^5,23–53^ studies have corroborated this interpretation of radiative heat transfer between structures of varied morphologies. This subject has generated fundamental insights that include important corrections due to nonlocal^30^, phonon^27,42^, and photonic band^47^ effects, as well as magnetic polarization^34^. Additionally, retardation, radiation emission, and crossed electric-magnetic terms in the optical response have been shown to severely modify the transfer power^50^. However, the so far observed and predicted transfer rates are slow compared with dissipative transport through the surrounding media, in which heat can cause undesired effects. This situation persists even when the interaction between neighboring structures is enhanced due to strong resonant excitations, such as plasmons in noble metals.

In this context, graphene plasmons can be advantageous because their frequencies lie in the mid-infrared, which is the spectral region for thermal interactions under attainable temperatures. Indeed, plasmon energies in graphene nanostructures scale as $\sim \sqrt{{E}_{F}/D}$ with the Fermi energy *E*_F_ and the characteristic size *D* (e.g., the diameter for a disk). Doping levels as high as *E*_F_ ∼ 1 eV have been reported through electrostatic gating^54^, and even higher values through chemical doping^55,56^, manifesting themselves in the opening of a 2*E*_F_ gap for vertical optical transitions^54,57^. However, plasmons are only well defined at energies below ∼ *E*_F_ due to the narrowing of the gap as their momentum increases^58^. For reference, a 20 nm disk supported on silica and doped to *E*_F_ = 1 eV exhibits a dipolar plasmon at ≈ 0.4 eV (ref. 58). This explains why experiments have only explored mid-infrared plasmons, as higher energies require smaller structures, whose fabrication can be challenging.

An additional advantage of graphene lies in its large electrical tunability, which enables an active control of these phenomena. In a related context, electrical modulation of thermal emission of radiation has been accomplished in gated nanostructured graphene^59^, while an optical-to-thermal converter has been proposed to be capable of efficiently transforming an optical pump into light emitted at longer mid-infrared wavelengths^60^. Electrical control of radiative heat transfer between graphene-coated surfaces or between extended graphene and other materials has been also proposed^61–65^.

The competing mechanism (relaxation into phonons) was initially thought to be rather slow in graphene^66^ (nanosecond scale), a prediction that was subsequently corrected to much shorter timescales (picoseconds) due coupling of hot charge carriers to optical phonons^67^, and so-called supercollision cooling^68^. The latter is consistent with experimental observations^69,70^. Recent calculations have also identified a remarkably fast rate of radiative transfer between graphene films^62,71^, graphene nanoribbons^72^, and extended heterostructures of graphene and hexagonal boron nitride (BN)^73^, although all of them involve picosecond or even longer timescales. However, we need much faster transfer rates in order to prevent most of the electronic heat from being absorbed into phonons. We accomplish such a goal in this paper by resorting to graphene nanostructures capable of sustaining plasmons within an energy range that is commensurate with *k*_B_ *T*. Incidentally, radiative energy transfer from graphene electrons to optical phonons in a silica substrate has been argued to explain the measured saturation of conductivity in the carbon layer and provide a viable way of observing quantum friction^74^.

Here, we exploit the extraordinary optical and thermal properties of graphene to show that ultrafast radiative heat transfer can take place between neighboring nanoislands. The commonly accepted scheme for dissipation of the thermal energy produced by electronic and optical inelastic losses (i.e., energy transfer to valence and conduction electrons of the system, followed by relaxation into phonons and subsequent heat flow into the surrounding media) is here challenged by the radiative transfer mechanism taking place between neighboring structures within femtosecond timescales, thus overcoming electron relaxation into the atomic lattice. Using attainable graphene nanostructure designs, we find that ultrafast radiative heat transfer produces thermalization of two neighboring islands that results in >50% of the electronic heat of the hot one being radiatively transferred to its colder neighbor. This extraordinary phenomenon is made possible by the large plasmonic field concentration that mediates the coupling between the neighboring graphene structures, as well as by the low-specific electronic heat of this material^58^. Additionally, plasmons in this material exhibit unprecedentedly large electrical tunability accompanied by strong confinement of the measured fields^75,76^, which have recently enabled high mid-infrared sensitivity in the detection of proteins^77^ and other organic molecules^78^. In a similar fashion, the ultrafast radiative heat transfer phenomenon here investigated can be actively switched on and off by gating the graphene structures.

## Results

### Radiative heat transfer between graphene nanodisks

**Fig. 1:**
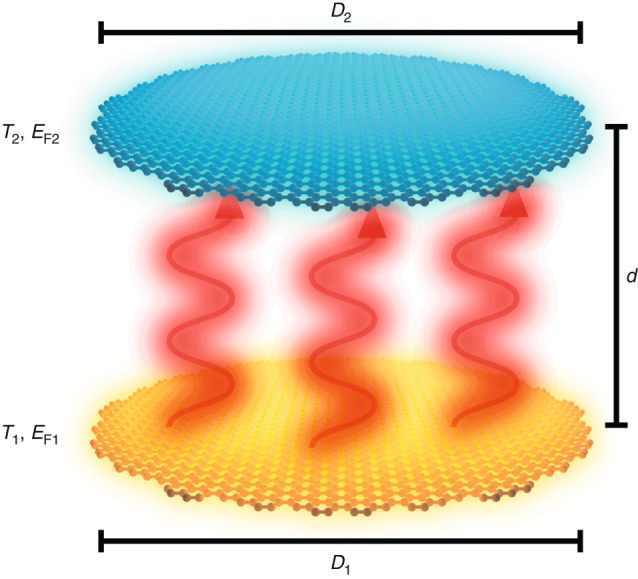
**Sketch of the structure considered for ultrafast radiative heat transfer.** We study heat transfer between two parallel coaxial graphene disks placed in vacuum and separated by a small distance *d*. Each disk $\ell \mathrm{=1,2}$ is characterized by its diameter ${D}_{\ell }$, Fermi energy ${E}_{{\rm{F}}\ell }$, and electron temperature ${T}_{\ell }$, with *T*_1_ > *T*_2_

We focus on the system depicted in Fig. 1, consisting of two parallel coaxial graphene nanodisks of diameters *D*_1_ and *D*_2_, separated by a distance *d* between carbon planes, doped to Fermi levels *E*_F1_ and *E*_F2_, and having electronic temperatures *T*_1_ > *T*_2_. For simplicity, we consider the disks to be placed in vacuum, as the conclusions of this work remain the same when the disks are surrounded by a dielectric material such as BN (e.g., *ϵ* ∼ 3.2, see Supplementary Fig. 1). Heat is radiatively transferred from the hotter disk to the colder one as a result of thermal fluctuations in both disks, whose interaction is mediated by their self-consistent electromagnetic response. In fact, for the small size of the structures under consideration compared with the thermal wavelengths ${\lambda }_{{T}_{\ell }}$ (with $\ell \mathrm{=1,2}$), retardation and magnetic response effects can be dismissed, so we only need to deal with charge fluctuations and their Coulomb interaction.

We calculate the heat transfer power (HTP) as the net balance of the work done by the thermally fluctuating charges of the hotter disk on the colder one minus the work done on the former by the fluctuating charges of the latter. This leads to a classical electromagnetic expression involving thermal fluctuations, which are evaluated by means of the fluctuation-dissipation theorem^79,80^. A detailed self-contained derivation is offered in the Methods section, leading to a compact expression [Eq. (8)] that is proportional to the integral over the exchanged frequency *ω*. The integrand consists of the difference between the Bose-Einstein occupation numbers ${n}_{\ell }={[\exp (\hbar \omega /{k}_{{\rm{B}}}{T}_{\ell })-1]}^{-1}$ of the two disks at their respective temperatures ${T}_{\ell }$, multiplied by a loss function that is determined by the disk susceptibilities ${\chi }_{\ell }$. The latter are dominated by plasmonic modes, which allow us to formulate a description in terms of plasmon wave functions (PWFs)^81,82^. Only the lowest-order PWFs contribute significantly to the HTP for the range of geometrical parameters under consideration. Their explicit form (see Methods), as well as full details on the PWF-based susceptibilities, are given in the Methods section. For coaxial disks (Fig. 1), we find that modes of different azimuthal number *m* do not mix, so we can separate their contributions to the HTP received by disk 2 as

1 $${P}_{2}=	\frac{2\hbar }{\pi }\sum _{m\mathrm{=0}}^{\infty }(2-{\delta }_{m0})\\ 	{\int }_{\!\!\!\!0}^{\infty }\omega {\rm d}\omega ({n}_{1}-{n}_{2}){\rm{Tr}}[{\Delta }^{m\dagger }\cdot {v}^{m}\cdot {\rm{Im}}\{{\chi }_{1}^{m}\}\cdot {v}^{m}\cdot {\Delta }^{m}\cdot {\rm{Im}}\{{\chi }_{2}^{m}\}]$$

(and also *P*_1_ = −*P*_2_), where Tr[…] stands for the trace, the matrix ${\Delta }^{m}={({\Bbb{I}}-{\chi }_{2}^{m}\cdot {v}^{m}\cdot {\chi }_{1}^{m}\cdot {v}^{m})}^{-1}$ accounts for multiple scattering between the disks, *v* ^*m*^ describes their mutual Coulomb interaction, and 𝕀 is a unit matrix. The matrices *v* ^*m*^ and ${\chi }_{\ell }^{m}$ contain elements projected on the PWFs with *m* azimuthal symmetry (see Methods for detailed expressions). Incidentally, the leading (2−*δ*_*m*0_) factor reflects the fact that *m* and −*m* modes yield the same contribution.

In this formalism, the optical response of graphene is described through its surface conductivity *σ*, for which we adopt the local-random-phase-approximation (local-RPA) model^58,83,84^ [see Eq. (25) in the Methods section]. We remark that, besides the explicit dependence of ${n}_{\ell }$ on ${T}_{\ell }$, the temperature enters *σ* through the chemical potential as well (see Methods). It should be noted that, in contrast to extended graphene, the lack of translational invariance in nanostructures prevents us from using the full nonlocal RPA conductivity^85,86^. However, a full RPA description of the optical absorption of the system under consideration based on a previous implementation for finite structures^87^ reveals that nonlocal effects only play a small role (see Supplementary Fig. 2). We further analyze heat transfer between closely spaced extended graphene films, and more specifically, the contribution coming from parallel wave-vector components $\sim 2\pi /{D}_{\ell }$, for which we find that nonlocal effects are also small for the graphene parameters under consideration (see Supplementary Note 4 and Supplementary Fig. 4), and therefore, we also expect them to be small for disks of diameter ${D}_{\ell }$.

Incidentally, as the HTP of Eq. (1) is an integrated quantity, it is not too sensitive to the model used for the graphene conductivity *σ*. This is corroborated in Supplementary Fig. 3a, b, where we compare results obtained using either the local-RPA or the Drude model [Eq. (25) with the *E* integral set to zero]. Only small discrepancies between the two models are observed at small separations *d* in the resulting HTP. Actually, the small *d* region is most sensitive to elements of the formalism such as the inclusion of multiple scattering in the optical response of the disks [Δ^*m*^ matrices in Eq. (1), see Supplementary Fig. 5 for a comparison with results obtained by setting Δ^*m*^ = 𝕀]. We also observe a mild dependence of the HTP on the value of the intrinsic electronic decay time (Supplementary Fig. 6), which we set to $\hbar {\tau }^{-1}\mathrm{=10}\,$ meV throughout this work. Additionally, we find good convergence of the HTP with the number of *m*’s and PWFs used in the calculations (Supplementary Fig. 7).

We stress that the relatively high temperatures under consideration (thousands of degrees) refer to the electronic gas of the material, which can be reached by optical pumping in the ultrafast regime^88–90^.

**Fig. 2:**
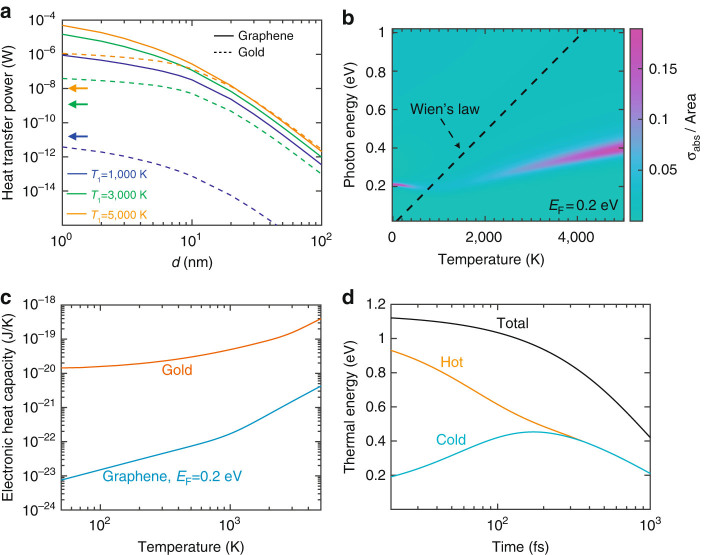
**Thermal and optical properties associated with radiative heat transfer. a** Dependence of the radiative heat transfer power (*HTP*) on the separation distance *d* between two graphene nanodisks (*solid curves*) compared with two gold nanodisks (*dashed curves*, disk thickness *t* = 2 nm). All disks are 20 nm in diameter. The HTP is plotted for different values of *T*_1_ (see legend), while the cold disk is at ambient temperature *T*_2_ = 300 K. The *arrows* indicate the HTP between two blackbodies of an area equal to that of the present disks and placed at temperatures *T*_1_ and *T*_2_. Both graphene nanodisks are assumed to be doped with the same Fermi energy *E*_F1_ = *E*_F2_ = 0.2 eV and described by the local-RPA conductivity (see Methods). **b** Optical absorption cross-section *σ*_abs_ normalized to the graphene area for one of the graphene disks considered in **a** as a function of photon energy $\hbar \omega$ and temperature *T*. The *dashed line* corresponds to Wien’s law, $\hbar \omega \approx 2.82\,{k}_{{\rm{B}}}T$. **c** Temperature dependence of the electronic heat capacity for one of the graphene (*blue curve*, see Methods) and gold (*red curve*, taken from ref. 91) nanodisks considered in **a**. **d** Illustrative example of the femtosecond dynamics of the electronic thermal energy in two graphene nanodisks under the conditions of **a** for a separation *d* = 1 nm, with initial temperatures *T*_1_ = 1,000 K and *T*_2_ = 300 K. The electronic thermal energy is shown for both the initially hot (*orange curve*) and cold (*cyan curve*) nanodisks, as well as their sum (*black curve*)

The disk separation dependence of the HTP is studied in Fig. 2a (solid curves) for 20 nm graphene disks doped to a Fermi level *E*_F_ = 0.2 eV, with the hotter disk at different temperatures *T*_1_ (see labels) and the colder one at room temperature *T*_2_ = 300 K. In general, higher temperatures *T*_1_ lead to larger HTP, due in part to the (*n*_1_−*n*_2_) factor in Eq. (1). At large separations $d\gg {D}_{\ell }$, only dipole–dipole interactions between the disks contribute efficiently to the transfer, leading to a 1/*d* ^6^ dependence, in agreement with the asymptotic expression of the electrostatic Eq. (9) (see Methods). A smooth convergence of the full calculation [Eq. (1)] to this limit [Eq. (9)] is observed in the additional calculations presented in Supplementary Fig. 8. The near-field character of heat transfer is further emphasized by considering the extension of the dominant dipole plasmon away from the disks (i.e., the electric-field amplitude decays by 1/*e* over a distance ∼ *D*/2*π*, as estimated from the out-of-plane decay of plasmons in extended graphene for an equivalent wavelength ∼ *D*), which explains the low slope in the curves of Fig. 2a at small *d*’s.

As a reference, we compare these results with the HTP for gold disks of the same diameter (Fig. 2a, broken curves), which we describe through an effective surface conductivity obtained from the measured dielectric function^92^ *ϵ*_Au_ as *σ*_Au_ = *iωt*(1−ϵ_Au_)/4*π*, where we take a thickness *t* = 2 nm. This approximation, which is reasonable because we are considering a small value of *t* compared with the diameter (20 nm), allows us to apply the same formalism as for graphene [Eq. (1)]. Despite the larger thickness of the gold disks, their HTP is much smaller than for graphene. In fact, plasmons in the graphene disks lie in the mid-infrared region for the parameters under consideration (i.e., their energies are commensurate with *k*_B_ *T*_1_), while those of the gold disks appear at much higher energies, and thus do not contribute efficiently to the heat transfer. This mismatch is partly alleviated at the highest temperature under consideration (*T*_1_ = 5,000 K), for which gold and graphene disks exhibit similar HTPs in the large *d* limit.

As an additional comparison, the left arrows in Fig. 2a show an estimate obtained from the Stefan-Boltzmann law^32^ for radiative heat transfer between two blackbodies of an area equal to that of the present disks. As anticipated above, graphene outperforms blackbodies by several orders of magnitude.

The strength of their optical response influences the ability of the disks to transfer energy radiatively. This is examined in Fig. 2b, where we plot the absorption cross-section of one of the graphene disks considered in Fig. 2a. An intense plasmon feature is observed in the 0.2–0.4 eV region, whose temperature dependence is inherited from the conductivity [Eq. (25)]. The dashed line in Fig. 2b shows the relation between the temperature and the photon energy according to Wien’s law (i.e., the value of $\hbar \omega$ at the maximum of ${\omega }^{3}{n}_{\ell }(\omega )$ as a function of ${T}_{\ell }$). This is relevant for the analysis of Eq. (1), in which a factor $\omega \,{n}_{\ell }(\omega )$ appears explicitly, whereas the remaining *ω* ^2^ factor comes from the low *ω* limit of the ${\rm{Im}}\{{\chi }_{\ell }^{m}\}$ matrices [obviously, the full *ω* dependence of the integrand of Eq. (1) is more complex, as shown in Supplementary Fig. 3g, h, but an analysis based on Wien’s law is still informative]. Additionally, the response functions entering the trace in Eq. (1) display maxima near the plasmons, and therefore, the overlap between the dashed line and the plasmon in Fig. 2b indicates that this excitation contributes efficiently to the HTP, thus providing a criterium for optimization. Incidentally, the plasmon dispersion and strength follow nonmonotonic behaviors resulting from the complex interplay between the increase in both the density of free charge carriers and the number of decay channels associated with single-electron transitions.

The electronic heat capacity provides a relation between the temperature and the amount of energy strored in the electron gas. In this respect, graphene is also advantageous relative to traditional plasmonic materials such as gold^91^ because its heat capacity is orders of magnitude smaller (Fig. 2c) as a result of its conical band structure, in contrast to the parabolic dispersion of gold conduction electrons. In consequence, cooling the graphene electrons requires transferring a smaller amount of heat, thus making the process potentially faster.

### Ultrafast radiative heat transfer regime

We study the heat transfer dynamics by considering the electronic heat ${Q}_{\ell }$ deposited on each graphene disk $\ell$ and the evolution of these quantities according to the equations

2 $${\dot{Q}}_{\ell }=-{\tau }_{{\rm{ph}}}^{-1}{Q}_{\ell }+{P}_{\ell },\quad \quad (\ell \mathrm{=1,2})$$

where ${P}_{\ell }$ are the transfer powers given by Eq. (1), while *τ*_ph_ is a phenomenological electron relaxation time (to phonons) that we approximate as 1 ps, a value of the order of what is observed in pump-probe experiments^67,93^. We note that the electronic heat of each disk $\ell$ depends on the electronic temperature ${T}_{\ell }$ as ${Q}_{\ell }=\beta \pi {D}_{\ell }^{2}{({k}_{{\rm{B}}}{T}_{\ell })}^{3}/{(2\hbar {v}_{{\rm{F}}})}^{2}$ [see Eq. (23) in the Methods section]. Also, the transfer powers *P*_1_ and *P*_2_ = −*P*_1_ [Eq. (1)] implicitly depend on both temperatures *T*_1_ and *T*_2_. In order to make this clearer, we provide equations equivalent to Eq. (2) at the end of the Methods section with a more explicit dependence on the temperatures, along with details of the numerical solution method. It should be pointed out that, because the electronic heat capacity in graphene is much smaller than that associated with the lattice, the temperature reached by the system when electrons and phonons are in thermal equilibrium is much smaller than the electron temperatures here considered after optical pumping. For this reason, we neglect the lattice in our analysis.

As an illustrative example, we show in Fig. 2d the evolution of ${Q}_{\ell }$ according to Eq. (2) for the two graphene disks considered in Fig. 2a when they are prepared at initial temperatures *T*_1_ = 1,000 K and *T*_1_ = 300 K: the cold disk more than doubles its electronic energy after ∼ 200 fs of evolution (peak of cyan curve), when it has gained nearly the same amount of energy as the one dissipated to the atomic lattice (decay of black curve). Notably, the disks reach mutual thermal equilibrium after only ∼ 250 fs, well before full relaxation takes place.

**Fig. 3:**
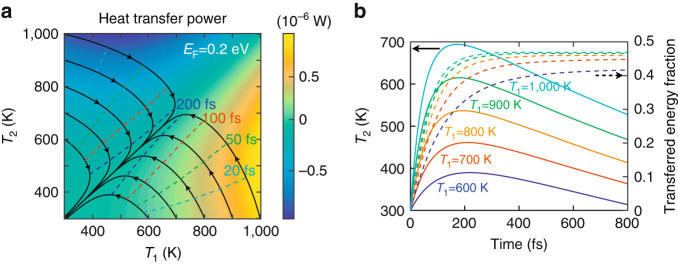
**Temperature and temporal dependences of radiative heat transfer. a** HTP between two graphene nanodisks (20 nm diameter, 0.2 eV Fermi energy, *d* = 1 nm separation) as a function of *T*_1_ and *T*_2_ for the geometry of Fig. 1. *Black solid curves* represent the evolution of the electron temperatures in the two nanodisks for different initial conditions. *Dashed curves* indicate the times (see labels) along the evolution of the *solid curves* from either the *vertical* or the *horizontal axes* of the plot. We assume an inelastic relaxation time (electron-lattice coupling) of 1 ps. **b** Temporal evolution of the electron temperature *T*_2_ in the colder disk (initially at *T*_2_ = 300 K, *left vertical scale*, *solid curves*) and the transferred energy fraction from disk 1 to disk 2 (*right vertical scale*, *dashed curves*) for different initial electron temperatures of the hotter disk *T*_1_ (see labels)

A more detailed study of the heat transfer dynamics is presented in Fig. 3 for 20 nm graphene disks separated a distance of 1 nm and doped to a Fermi energy of 0.2 eV. The color plot of Fig. 3a shows the HTP as a function of the temperatures in the two disks. Further calculations for a wider range of temperatures and more values of the disk diameters and the doping levels are presented in Supplementary Figs. 9 and 10. Obviously, the diagonal of this plot corresponds to zero transfer, when the two particles have the same temperature. The black solid curves represent the evolution of the disk temperatures starting from initial conditions at the plot axes (i.e., with one of the disks at 300 K and the other one at higher temperature). The evolution is along the direction of the arrows, with positions at specific times indicated by the dashed curves. Interestingly, the evolution toward the diagonal (thermal equilibrium) is characterized by a significant increase in the temperature of the colder disk (Δ*T* ∼ 400 K) within the first 100–200 fs, much faster than relaxation to the atomic lattice. This evolution involves the transfer of a large fraction of electronic heat to the colder disk, as shown in Fig. 3b: when the disks are prepared at 1,000 and 300 K initial temperatures, nearly 50% of the electronic heat of the hot disk is transferred to the cold one within the first ∼ 200 fs. We remark that fast transfers take place over a wide temperature range down to substantially smaller *T*′s (see Fig. 3a). These conclusions are maintained when considering larger disks (40 nm diameter) or wider separations (3 nm), as shown in Supplementary Fig. 11. They are also maintained when considering higher doping levels (Supplementary Fig. 12), well above the dipole plasmon energy, a condition for which nonlocal effects are particularly negligible. These supplementary figures also show that the results are robust with respect to variations in the disk diameters (e.g., similar conclusions are obtained for two dissimilar disks with diameters differing by a few nanometers).

**Fig. 4:**
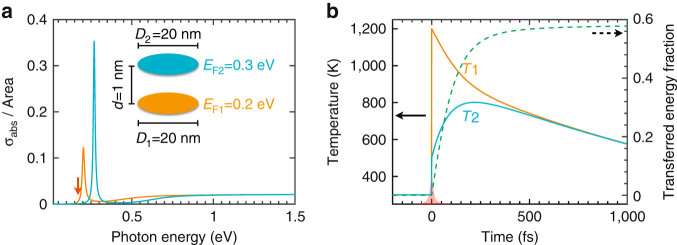
**Ultrafast radiative heat transfer induced by optical pumping. a** Normalized absorption cross-sections *σ*_abs_ of two graphene nanodisks (20 nm diameter, *d* = 1 nm separation) at the same initial temperature of 300 K but doped with different Fermi energies (see inset). **b** Time evolution of the electron temperatures *T*_1_ and *T*_2_ (*left vertical scale, solid curves*) and transferred energy fraction (*right vertical scale, dashed curve*) after optical pumping (150 mJ m^−2^ light fluence, 0.17 eV photon energy, as indicated by the *red arrow* of **a**)

In practical implementations, optical pumping with femtosecond laser pulses grants us access into the ultrafast regime, allowing us to reach high electron temperatures such as those considered in this work^94–96^. Additionally, the amount of optically absorbed energy depends on the pump frequency relative to the plasmons of the system^97^. This idea can be exploited to pump neighboring graphene disks in such a way that one of them absorbs much more energy than the other, just by tuning the pump laser near the plasmon of one of the disks and away from the plasmons of the other disk. We thus need disks of either different diameters or different Fermi levels. We consider the latter possibility, which can be realized in practice through the variation in intrinsic doping produced by an asymmetric dielectric environment, or also by creating different potential landscapes through an asymmetric doping geometry. The system under investigation is depicted in the inset of Fig. 4a: two 20 nm graphene disks, separated by 1 nm, initially placed at 300 K, and doped to Fermi energies 0.2 and 0.3 eV, respectively. We consider optical pumping at a photon energy of 0.17 eV with a fluence of 150 mJ m^−2^. The pulse energy is closer to the lower doping disk (Fig. 4a), and thus, this is the one that reaches a higher temperature. For simplicity, we assume instantaneous pumping (i.e., a *δ*-function temporal profile of the pulse), which rapidly elevates the electron temperatures to *T*_1_ ∼ 1,200 K and *T*_2_ ∼ 500 K (Fig. 4b, left end). Interestingly, although the plasmons in the two disks are off-resonance before irradiation, optical pumping produces a larger blue shift in the hotter disk, bringing it on resonance with the initially bluer plasmon of the colder disk. Ultrafast radiative heat transfer is again observed, leading to mutual equilibrium between the disks (*T*_1_ ≈ *T*_2_) within ∼ 500 fs, which is accompanied by nearly 60% of the electronic heat of disk 1 being transferred to disk 2. We remark that higher that 50% transferred energy fraction is made possible by the doping asymmetry, which directly affects the heat capacity (see Methods). An interesting question for future studies relates to the maximum energy fraction that can be transferred in optimized structures.

## Discussion

Our prediction of ultrafast radiative heat transfer in graphene provides a fundamentally unique scenario: radiative coupling is capable of evacuating electronic heat from a nanoisland to a surrounding structure fast enough to prevent substantial relaxation into the atomic lattice. This is accomplished with attainable geometrical and material parameters: tens of nanometers in lateral size *D* in structures that can be patterned through state-of-the-art lithography^77,98^ and bottom-up synthesis^99–101^; vertical separations of a few nanometers, as provided by van der Waals atomic layer spacers^102–104^; tenths of electronvolts Fermi energy *E*_F_, controllable through electrical gating^54,57^; and electron temperatures *T* of thousands of degrees reached by ultrafast optical pumping^88–90,105^.

Although we have focused on disks for computational convenience, we expect our conclusions to be maintained for other geometries of similar lateral size because the HTP is a frequency-integrated quantity that should be qualitatively independent of the actual spectral position of the plasmon modes, as long as they overlap with Wien’s law (see Fig. 2b) and they are highly correlated with each other in the two islands. This correlation can be facilitated if the islands are nearly identical in shape and size. Actually, this is a condition that can be accomplished through lateral patterning of a stack formed by two graphene films and an atomically thin van der Waals layer spacer, using for example e-beam lithography.

In practice, the disks could have intrinsic doping due to interaction with a dielectric environment, which can change the Fermi energy by as much as ∼ 0.3 eV. Obviously, because the disks do not have electrical connectivity, their control through electrostatic gating presents a challenge. However, gating should be possible in a configuration consisting of neighboring graphene ribbons, which can be biased and exposed to distant gates. The contacts can be placed far from the ribbon region in which heat transfer takes place, while the gates can also be 100 s nm away and thus should not affect the heat transfer.

Our choice of parameters leads to graphene plasmon energies^58^ $\hbar {\omega }_{m\nu }\sim e\sqrt{{E}_{{\rm{F}}}/(-\pi {\eta }_{m\nu }D)}$ (as estimated from a Drude model description for the graphene conductivity, see Table 1 for values of the eigenvalue *η*_*mν*_ associated with disk plasmons) that are commensurate with *k*_B_ *T* (i.e., they overlap the broad spectral peak of thermal emission, see Supplementary Fig. 3). As a consequence, the characteristic time interval *τ*_RHT_ required to radiatively transfer a sizable fraction of the electronic heat energy is reduced to the femtosecond domain.

**Table 1:** Eigenvalues associated with the disk PWFs

*ν*/***m***	0	1	2	3	4	5
1	0.0234	0.0720	0.0402	0.0283	0.0220	0.0181
2	0.0123	0.0165	0.0130	0.0109	0.0094	0.0083
3	0.0084	0.0101	0.0086	0.0076	—	—
4	—	0.0073	—	—	—	—

**Fig. 5:**
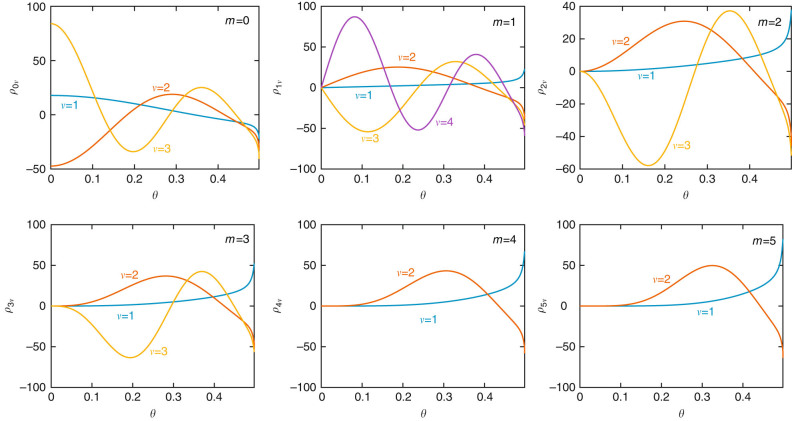
**Radial components of the disk PWFs.** We show *ρ*_*mν*_(*θ*) as defined in Eq. (16a) and Eq. (16b) for several low values of *m* and *ν* (see also Table 1)

We list the values of −*η*_*mν*_ corresponding to the disk PWFs *ρ*_*mν*_ considered in Fig. 5 [see Eq. (16a) and Eq. (16b) for *m*=0–5 and *ν*=1–4]

*PWFs* plasmon wave functions

A simple dimensional analysis reveals that the HTP is proportional to *E*_F_/*D*, provided the ratios of disk diameters and temperatures, as well as *d*/*D* and the quantity *E*_F_/*DT* ^2^, are kept constant (see also Supplementary Figs. 9 and 10). The optimum temperature at which maximum transfer takes place scales as $T\propto \sqrt{{E}_{{\rm{F}}}/D}$. Additionally, we find the scaling *τ*_RHT_ ∝ *E*_F_ *D* ^3^ with Fermi energy and lateral size, and therefore, low doping levels and small sizes enable faster cooling. These conclusions are consistent with the detailed numerical analysis of *τ*_RHT_ presented in Supplementary Fig. 13.

We stress that the formalism developed in the Methods section can be readily applied to study radiative coupling assisted by fluctuations of other types of excitations besides plasmons, such as optical phonons in two-dimensional polar materials, whose relative characteristic transfer time deserves further analysis.

Another interesting possibility consists in combining more than two structures. This could be used to accelerate the rate of heat evacuation and achieve greater control over the spatial flow of radiative heat transfer. Higher transfer rates could be also obtained through lateral shape optimization or by relying on other carbon allotropes such as carbon nanotubes. Additionally, similar fast transfers should be enabled by a wide range of existing atomic-scale materials capable of sustaining confined optical excitations^106^ (e.g., exciton polaritons in dichalcogenides). Besides the fundamental interest of this line of research, electronic cooling via radiative heat transfer constitutes a promising avenue to effectively suppress relaxation to the atomic lattice, thus preventing thermal damage in nanoscale devices.

## Methods

### Theory of radiative heat transfer

We consider two structures labeled by the index $\ell \mathrm{=1,}\,2$, each of them assumed to be in internal thermal equilibrium at a temperature ${T}_{\ell }$. Radiative heat transfer can take place if *T*_1 _≠ *T*_2_, mediated by electromagnetic interaction at characteristic frequencies $\sim {k}_{{\rm{B}}}{T}_{\ell }/\hbar$ ^50^. We further assume the corresponding light wavelengths $\sim 2\pi \hbar c/{k}_{{\rm{B}}}{T}_{\ell }$ to be much larger than the size of the structures. The response of the latter can be then described in the quasistatic limit through their susceptibilities ${\chi }_{\ell }({\bf{r}},{\bf{r}}{\prime} ,\omega )$, which are defined as the induced charge density distribution at **r** produced by a unit potential point source oscillating with frequency *ω* at **r**′. The charge density induced in the $\ell$ structure by a monochromatic potential $\phi ({\bf{r}})\exp (-i\omega t)+{\rm{c}}{\rm{.c}}{\rm{.}}$ is then given by $\int {\rm d}^{3}{\bf{r}}{\prime} {\chi }_{\ell }({\bf{r}},{\bf{r}}{\prime} ,\omega )\phi ({\bf{r}}{\prime} )\,\exp (-i\omega t)+{\rm{c}}{\rm{.c}}{\rm{.}}$ Incidentally, although the emission of radiation away from the system is not accounted for within the quasistatic limit, this is a negligible contribution for the small structures under consideration, in which radiative heat transfer and relaxation to the atomic lattice occur at a much faster rate.

We express the net power received by structure 2 as the work *P*_2←1_ done on 2 by charges fluctuating in 1 minus the work *P*_1←2_ done on 1 by charges fluctuating in 2. It is enough to calculate the latter in detail, because the former is simply obtained by interchanging the subindices 1 and 2 in the resulting expression. We start from *P*_1←2_ = −〈∫d^3^ **rj**_1_(**r**, *t*) ⋅∇*ϕ*_2_(**r**, *t*)〉, which is the work exerted by the electric field −∇*ϕ*_2_(**r**, *t*) produced by fluctuations in 2, acting on the current **j**_1_(**r**, *t*) of 1. Here, 〈…〉 denotes the average over thermal fluctuations, the space integral extends over the entire three-dimensional space, and the function **j**_1_ is a distribution that vanishes outside the graphene and may exhibit a singularity at the edge. Integrating the ∇ operator by parts, writing the electric potential *ϕ*_2_ in terms of the charge *ρ*_2_ *via* the Coulomb potential *v*(**r**, **r**′) (e.g., *v* = 1/ϵ|**r**−**r**′| in a homogeneous medium of permittivity ϵ), and using the continuity equation ∇⋅**j**_1_ = −∂_*t*_ *ρ*_1_, we find *P*_1←2_ = −〈∫*d* ^3^ **r** *d* ^3^ **r**′∂_*t*_(*ρ*_1_(**r**, *t*))*v*(**r**, **r**′)*ρ*_2_(**r**′, *t*)〉, or equivalently,

3 $${P}_{1\leftarrow 2}=	i\int\!\!\!\! \int \frac{{\rm d}\omega {\rm d}\omega {\prime} }{{(2\pi )}^{2}}\omega {{\rm{e}}}^{-i(\omega +\omega {\prime} )t}\left\langle \int {\rm d}^{3}{\bf{r}}{\rm d}^{3}{\bf{r}}{\prime} {\rho }_{1}({\bf{r}},\omega )v({\bf{r}},{\bf{r}}{'}){\rho }_{2}({\bf{r}}{'},\omega {\prime} )\right\rangle \\ 	=i\int\!\!\!\! \int \frac{{\rm d}\omega {\rm d}\omega {\prime} }{{(2\pi )}^{2}}\omega {{\rm{e}}}^{-i(\omega +\omega {\prime} )t}\langle {\rho }_{1}{(\omega )}^{{\rm{T}}}\cdot v\cdot {\rho }_{2}(\omega {\prime} )\rangle ,$$

where we have expressed the charges in frequency space *ω* and replaced ∂_*t*_ by −*iω*. The last line of Eq. (3) implicitly defines a matrix notation in which **r** and **r**′ are used as matrix indices, while the dot indicates matrix multiplication. In this notation, ${\rho }_{\ell }$ are column vectors, *v* and ${\chi }_{\ell }$ are matrices, and ${\rho }_{\ell }^{{\rm{T}}}$ is the transpose of ${\rho }_{\ell }$.

The self-consistent charges ${\rho }_{\ell }$ produced by the fluctuating charge ${\rho }_{2}^{{\rm{fl}}}$ are now obtained from the relations

$${\rho }_{1}={\chi }_{1}\cdot v\cdot {\rho }_{2},\ {\rho }_{2}={\chi }_{2}\cdot v\cdot {\rho }_{1}+{\rho }_{2}^{{\rm{fl}}},$$

where we work in the frequency domain and use the matrix notation introduced above. We remark that ${\rho }_{2}^{{\rm{fl}}}({\bf{r}},\omega )$ vanishes for **r** outside structure 2, while ${\chi }_{\ell }({\bf{r}},{\bf{r}}{\prime} ,\omega )$ vanishes for **r** or **r**′ outside $\ell$. By construction, *v*(**r**, **r**′) only needs to be evaluated for **r** and **r**′ sitting at different structures. Inserting the solution of these equations into Eq. (3), we find

4 $${P}_{1\leftarrow 2}=	i\int\!\!\!\! \int \frac{{\rm d}\omega {\rm d}\omega {\prime} }{{(2\pi )}^{2}}\omega {{\rm{e}}}^{-i(\omega +\omega {\prime} )t}\\ 	\times \int {\rm d}^{3}{\bf{r}}\int {\rm d}^{3}{\bf{r}}{\prime} \langle [{\chi }_{1}(\omega )\cdot v\cdot \Delta (\omega )\cdot {\rho }_{2}^{{\rm{fl}}}(\omega )]{|}_{{\bf{r}}}v({\bf{r}},{\bf{r}}{\prime} )[\Delta (\omega {\prime} )\cdot {\rho }_{2}^{{\rm{fl}}}(\omega {\prime} )]{|}_{{\bf{r}}{\prime} }\rangle ,$$

where

5 $$\Delta ={({\Bbb{I}}-{\chi }_{2}\cdot v\cdot {\chi }_{1}\cdot v)}^{-1},$$

whereas 𝕀 is the unit matrix (i.e., *δ*(**r**−**r**′)). Now, the average over thermal fluctuations can be carried out using the fluctuation-dissipation theorem^79,80,107,108^

6 $$\langle {\rho }_{\ell }^{{\rm{fl}}}({\bf{r}},\omega ){\rho }_{\ell {\prime} }^{{\rm{fl}}}({\bf{r}}{\prime} ,\omega {\prime} )\rangle =-4\pi \hbar {\delta }_{\ell \ell {\prime} }\delta (\omega +\omega {\prime} )[{n}_{\ell }(\omega )+\mathrm{1/2}]{\rm{Im}}\{{\chi }_{\ell }({\bf{r}},{\bf{r}}{\prime} ,\omega )\},$$

where ${n}_{\ell }(\omega )={[\exp (\hbar \omega /{k}_{{\rm{B}}}{T}_{\ell })-1]}^{-1}$ is the Bose-Einstein distribution at temperature ${T}_{\ell }$ (i.e., for structure $\ell$). A detailed self-contained derivation of Eq. (6) is offered in the Supplementary Note 1. We find Eq. (4) to reduce to

7 $${P}_{1\leftarrow 2}=\frac{2\hbar }{\pi }{\int }_{\!\!\!\!0}^{\infty }\omega \,{\rm d}\omega ({n}_{2}+\mathrm{1/2})\,{\rm{Tr}}[{\Delta }^{\dagger }\cdot v\cdot {\rm{Im}}\{{\chi }_{1}\}\cdot v\cdot \Delta \cdot {\rm{Im}}\{{\chi }_{2}\}],$$

where Tr[…] stands for the trace, † refers to the conjugate transpose, and a dependence on *ω* is understood in all quatities. In the derivation of Eq. (7), we have used the properties *v* = *v* ^T^ and ${\chi }_{\ell }={\chi }_{\ell }^{{\rm{T}}}$ (reciprocity), ${\chi }_{\ell }(\omega )={\chi }_{\ell }^{\ast }(-\omega )$ (causality), $[{n}_{\ell }(\omega )+\mathrm{1/2}]=-[{n}_{\ell }(-\omega )+\mathrm{1/2}]$, Tr[*A*] = Tr[*A* ^T^], and Tr[*A*⋅*B*] = Tr[*B*⋅*A*] (see Supplementary Note 2 for further details).

Finally, the net power received by 2 is obtained from

8 $${P}_{2}={P}_{2\leftarrow 1}-{P}_{1\leftarrow 2}=\frac{2\hbar }{\pi }{\int }_{\!\!\!\!0}^{\infty }\omega \,{\rm d}\omega ({n}_{1}-{n}_{2})\,{\rm{Tr}}[{\Delta }^{\dagger }\cdot v\cdot {\rm{Im}}\{{\chi }_{1}\}\cdot v\cdot \Delta \cdot {\rm{Im}}\{{\chi }_{2}\}],$$

where the matrix Δ [see Eq. (5)] accounts for multiple scattering between the two structures. Incidentally, the latter cannot be ignored at short separations, as shown in Supplementary Fig. 5. From the invariance of the expression in the square brackets of Eq. (8) under exchange of the subindices 1 and 2 (see Supplementary Note 2), we confirm the expected result *P*_1_ = −*P*_2_.

Finally, for structures separated by a large distance *d* compared to their sizes, in virtue of induced-charge neutrality (i.e., $\int {\rm d}^{3}{\bf{r}}\,{\chi }_{\ell }({\bf{r}},{\bf{r}}{\prime} ,\omega )\mathrm{=0}$ for each $\ell$), the leading contribution to *v* is the dipole–dipole interaction. For parallel disks placed in vacuum, like the ones considered throughout this work, neglecting multiple scattering (i.e., taking Δ = 𝕀), we find from Eq. (8)

9 $${P}_{2}\approx \frac{4\hbar }{\pi {d}^{6}}{\int }_{\!\!\!\!0}^{\infty }\omega \,{\rm d}\omega \,({n}_{1}-{n}_{2})\,{\rm{Im}}\{{\alpha }_{1}\}{\rm{Im}}\,\{{\alpha }_{2}\},$$

where

10 $${\alpha }_{\ell }(\omega )=-\int x\,{\rm d}^{3}{\bf{r}}\int x{\prime} \,{\rm d}^{3}{\bf{r}}{\prime} {\chi }_{\ell }({\bf{r}},{\bf{r}}{\prime} ,\omega )$$

is the polarizability of disk $\ell$ along a direction *x* parallel to it. An extra factor of 2 has been introduced in Eq. (9) to account for the two equivalent orthogonal directions in the planes of the disks. The convergence of Eq. (8) toward Eq. (9) is illustrated by calculations presented in Supplementary Fig. 8.

### Description of graphene islands through PWFs

We now apply the above formalism to two parallel graphene islands placed in a homogeneous medium of permittivity ϵ and separated by a vertical distance $d=|{z}_{\ell }-{z}_{\ell {\prime} }|$ along their normal direction *z*. It is then convenient to use an eigenmode expansion for the response of each island $\ell$ ^81,82^. This allows us to define a complete set of PWFs ${\rho }_{\ell j}$ and real eigenvalues ${\eta }_{\ell j}$, where *j* is a mode index. More precisely, the susceptibility of the $\ell$ island, taken to be in the $z={z}_{\ell }$ plane, admits the rigorous exact expansion^81^

11 $${\chi }_{\ell }({\bf{r}},{\bf{r}}{\prime} ,\omega )=\frac{\epsilon }{{D}_{\ell }^{3}}\sum _{j}\frac{{\rho }_{\ell j}(\overrightarrow{\theta }){\rho }_{\ell j}(\overrightarrow{\theta }{\prime} )}{\mathrm{1/}{\eta }_{\ell j}-\mathrm{1/}{\eta }^{(\ell )}(\omega )}\,\delta (z-{z}_{\ell })\delta (z{\prime} -{z}_{\ell }),$$

where *j* runs over eigenmodes, we use the notation ${\bf{r}}=({D}_{\ell }\overrightarrow{\theta },z)$, $\overrightarrow{\theta }$ is an in-plane coordinate vector normalized to a characteristic length of the structure ${D}_{\ell }$ (we use the diameter for disks), and

12 $${\eta }^{(\ell )}(\omega )=\frac{i{\sigma }_{\ell }(\omega )}{\epsilon \omega {D}_{\ell }}$$

incorporates the response of the graphene through its local conductivity ${\sigma }_{\ell }(\omega )$. It should be noted that the latter depends on $\ell$ *via* the level of doping and the temperature (see below). The PWFs and their eigenvalues satisfy the orthogonality relation^81^

13 $$\int {\rm d}^{2}\overrightarrow{\theta }\int {\rm d}^{2}\overrightarrow{\theta }{\!\prime} \,\frac{{\rho }_{\ell j}(\overrightarrow{\theta }){\rho }_{\ell j{\prime} }(\overrightarrow{\theta }{\!\prime} )}{|\overrightarrow{\theta }-\overrightarrow{\theta }{\!\prime} |}=-\frac{{\delta }_{jj{\prime} }}{{\eta }_{\ell j}}\mathrm{.}$$

For islands with the same geometrical shape (e.g., disks), the PWFs and eigenvalues are independent of size ${D}_{\ell }$, even if *D*_1_ ≠ *D*_2_.

We can readily use Eq. (11) to evaluate the heat transfer rate according to Eq. (8). With some straightforward redefinitions, these equations remain the same, but now the coefficients of the matrices that they contain are labeled by eigenmode indices *j* instead of spatial coordinates **r**. More precisely, ${\chi }_{\ell }$ becomes a diagonal matrix of coefficients

$${\chi }_{\ell ,jj{\prime} }={\delta }_{jj{\prime} }\,\frac{\epsilon }{{D}_{\ell }^{3}}\frac{1}{\mathrm{1/}{\eta }_{\ell j}-\mathrm{1/}{\eta }^{(\ell )}},$$

while the matrix elements of the Coulomb interaction reduce to

14 $${v}_{jj{\prime} }=\frac{{D}_{\ell }^{2}{D}_{\ell {\prime} }^{2}}{\varepsilon }\int {\rm d}^{2}\overrightarrow{\theta }\int {\rm d}^{2}\overrightarrow{\theta }{\!\prime} \,\frac{{\rho }_{\ell j}(\overrightarrow{\theta }){\rho }_{\ell {\prime} j{\prime} }(\overrightarrow{\theta }{\!\prime} )}{\sqrt{{|{D}_{\ell }\overrightarrow{\theta }-{D}_{\ell {\prime} }\overrightarrow{\theta }{\!\prime} |}^{2}+{d}^{2}}}$$

when the operators to the left and right of *v* are referred to islands $\ell$ and $\ell {\prime}$, respectively. Incidentally, in this work we focus on disk dimers that share the same axis of symmetry; an eventual lateral displacement **b** between the islands is however easy to implement by adding it to ${D}_{\ell }\overrightarrow{\theta }-{D}_{\ell {\prime} }\overrightarrow{\theta }{\!\prime}$ in the above expression.

In this PWF formalism, inserting Eq. (11) into Eq. (10), we find that the polarizability of a graphene island along a given in-plane symmetry direction *x* is given by

15 $${\alpha }_{\ell }(\omega )=\epsilon {D}_{\ell }^{3}\sum _{j}\frac{{\zeta }_{j}^{2}}{\mathrm{1/}{\eta }^{(\ell )}-\mathrm{1/}{\eta }_{j}},$$

where ${\zeta }_{j}=\int {\theta }_{x}{\rm d}^{2}\overrightarrow{\theta }{\rho }_{j}(\overrightarrow{\theta })$ is a normalized plasmon dipole moment.

### PWFs for disks

In the disk geometry, the azimuthal number *m* provides a natural way of classifying the PWFs. More precisely, we can label them using a double index (*mν*) and separate the radial and azimuthal dependences as

16a $${\rho }_{m\nu }^{{\rm{c}}}(\overrightarrow{\theta })={\rho }_{m\nu }(\theta )\cos (m{\varphi }_{\overrightarrow{\theta }}),\quad (m\ge 0),$$

16b $${\rho }_{m\nu }^{{\rm{s}}}(\overrightarrow{\theta })={\rho }_{m\nu }(\theta )\sin (m{\varphi }_{\overrightarrow{\theta }}),\quad (m\ge 1).$$

We insist that these PWFs are the same for both disks in a dimer, as they are independent of disk size, and therefore, we drop the disk index $\ell$ for them. We also note that the PWFs are doubly degenerate for *m* > 0 (i.e., they share the same eigenvalue *η*_*mν*_ and radial component *ρ*_*mν*_(*θ*) for both sine and cosine azimuthal dependences). We obtain the radial component *ρ*_*mν*_(*θ*) by solving the Maxwell equations numerically using the boundary-element method^109^ for a self-standing disk of small thickness *t* ∼ *D*/100 compared with its diameter *D*. The disk is described by a dielectric function ϵ = 1 + 4*πiσ*/*ωt*, where *σ* is the Drude graphene conductivity (the actual model used for *σ* is irrelevant, as the PWFs depend only on geometry and not on the specifics of the material). In the limit of small damping, the plasmons emerge as sharp, spectrally isolated features in the local density of optical states (LDOS)^110^. We average the LDOS over a set of off-center locations in order to access different *m*’s efficiently. The radial components of the PWFs are then retrieved from the induced charge density, while the eigenvalues are derived from the resonance condition *η*_*mν*_ = Re{*iσ*/*ωD*} at the corresponding LDOS peak maximum.

By construction, ${\rho }_{m\nu }^{{\rm{c}}}$ and ${\rho }_{m\nu }^{{\rm{s}}}$ [see Eq.(16a) and Eq. (16b)] are mutually orthogonal according to Eq. (13). Additionally, PWFs with different *m*’s are automatically orthogonal. For the remaining pairs of wave functions that share both the value of *m* and the azimuthal dependence (either sine or cosine), Eq. (13) reduces to

17 $$-2\pi (1+ \delta_{m,0})\sqrt{{\eta }_{m\nu }{\eta }_{m\nu {\prime} }}{\int }_{\!\!\!\!0}^{\mathrm{1/2}}\theta \,{\rm d}\theta \,{\rho }_{m\nu }(\theta )\,{\int }_{\!\!\!\!0}^{\mathrm{1/2}}\theta {\prime} {\rm d}\theta {\prime} \,{\rho }_{m\nu {\prime} }(\theta {\prime} )\\ {\int }_{\!\!\!\!0}^{\pi }{\rm d}\varphi \,\frac{\cos (m\varphi )}{\sqrt{{\theta }^{2}+{\theta {\prime} }^{2}-2\theta \theta {\prime} \cos \,\varphi }}={\delta }_{\nu \nu {\prime} }\mathrm{.}$$

Our calculated radial PWFs, already normalized according to Eq. (17), are shown in Fig. 5 for the lowest values of (*mν*), while their associated eigenvalues are given in Table 1. The orthogonality for *ν* ≠ *ν*′ is rather satisfactory, as illustrated in Table 2, which shows the values obtained by numerically evaluating the left-hand side of Eq. (17).

**Table 2:** Orthogonality of the disk PWFs

	*m* = 0		*m* = 1			*m* = 2		*m* = 3		*m* = 4	*m* = 5
*ν*/*ν*′	1	2	1	2	3	1	2	1	2	1	1
2	0.008	1	0.055	1	—	0.058	1	0.061	1	0.063	0.064
3	0.006	0.010	0.114	−0.031	1	0.113	−0.028	0.114	−0.026	—	—
4	—	—	0.078	−0.019	−0.026	—	—	—	—	—	—

Each entry in this table is obtained by numerically integrating the left-hand side of Eq. (17). The values of *m*, *ν*, and *ν*′ cover the ranges considered in Fig. 5 and Table 1. All diagonal entries (*ν* = *ν*′) are 1 by construction. We only show *ν*≥*ν*′ values because the results are invariant under exchange of these two indices

*PWFs* plasmon wave functions

Upon insertion of the disk PWFs in Eq. (14), we find that *v*_*jj*′_ is diagonal by blocks (two blocks per *m*, corresponding to the two different azimuthal symmetries of Eq. (16a) and Eq. (16b) and each of them contributing the same to the HTP). As ${\chi }_{\ell ,jj{\prime} }$ is diagonal, this allows us to write *P*_2_ as a sum over *m*’s, essentially reflecting the fact that only modes of the same symmetry undergo mutual Coulomb interaction. The integrand of Eq. (8) then becomes an analytical function (see expressions for ${n}_{\ell }$, ${\chi }_{\ell }$, and Δ above), except for the integral over radial wave functions in *v*_*jj*′_, for which we derive a computationally convenient expression in Supplementary Note 3. We finally write Eq. (1) for the HTP, where the explicit dependence of the involved matrices on *m* is indicated.

Only *m* = 1 PWFs exhibit nonzero dipole moments *ζ*_*ν*_ contributing to the polarizability ${\alpha }_{\ell }$ in Eq. (15). More precisely, *ζ*_*ν*_ is 0.84, 0.40, 0.11, and 0.08 for *ν* = 1−4, respectively. We use these coefficients and Eq. (15) to obtain the absorption cross-section (Figs. 2b, 4a and Supplementary Fig. 3c–f) as

18 $${\sigma }_{\ell }^{{\rm{abs}}}(\omega )=\left(\frac{4\pi \omega }{c}\right){\rm{Im}}\{{\alpha }_{\ell }\}-\left(\frac{8\pi {\omega }^{4}}{3{c}^{4}}\right){|{\alpha }_{\ell }|}^{2},$$

where the second term ($\propto {|{\alpha }_{\ell }|}^{2}$) is negligible for the small diameters of the disks under consideration (≪ light wavelength).

### Temperature-dependent graphene chemical potential

At zero temperature, the Fermi energy *E*_F_ describes a charge-carrier doping density *n* subject to the relation^111^ ${E}_{{\rm{F}}}=\hbar {v}_{{\rm{F}}}\sqrt{\pi n}$, where *v*_F_ ≈ 10^6^ m s^−1^ is the Fermi velocity. This expression assumes a conical electronic band structure, which provides an accurate description for electron energies *E* up to a couple of electronvolts away from the Dirac point^112^. For concreteness, we consider doping with electrons, as exactly the same results are obtained when doping with holes within the conical band approximation. At finite temperature *T*, the population of electronic states is given by the Fermi-Dirac distribution

$${f}_{T}(E)=\frac{1}{{{\rm{e}}}^{(E-\mu )/{k}_{{\rm{B}}}T}+1},$$

where *μ* is the chemical potential. The latter depends on temperature in such a way that the electron density

19 $$n=\frac{4}{A}\sum _{{{\bf{k}}}_{\parallel }}[{f}_{T}(E)+{f}_{T}(-E)-1]$$

is maintained constant. Here, *A* is the graphene area, the factor of four originates in valley and spin degeneracies, ${{\bf{k}}}_{\parallel }$ is the parallel wave vector, $E=\hbar {v}_{{\rm{F}}}{k}_{\parallel }  >0$ is the electron energy in the upper Dirac cone, *f*_*T*_(*E*) is the electron population in that cone, and 1−*f*_*T*_(−*E*) is the hole distribution in the lower cone. Recasting the sum over ${{\bf{k}}}_{\parallel }$ into an integral (i.e., ${\sum }_{{{\bf{k}}}_{\parallel }}\to (A\mathrm{/2}\pi ){\int }_{\!0}^{\infty }{k}_{\parallel }{\rm d}{k}_{\parallel }$), and defining $x=\hbar {v}_{{\rm{F}}}{k}_{\parallel }/{k}_{{\rm{B}}}T$, Eq. (19) becomes

20 $${\left(\frac{{E}_{{\rm{F}}}}{{k}_{{\rm{B}}}T}\right)}^{\!\!2}\mathrm{=2}{\int }_{\!\!\!0}^{\infty }x{\rm d}x\,\left[\frac{1}{{{\rm{e}}}^{x-\mu /{k}_{{\rm{B}}}T}+1}-\frac{1}{{{\rm{e}}}^{x+\mu /{k}_{{\rm{B}}}T}+1}\right]\mathrm{.}$$

**Fig. 6:**
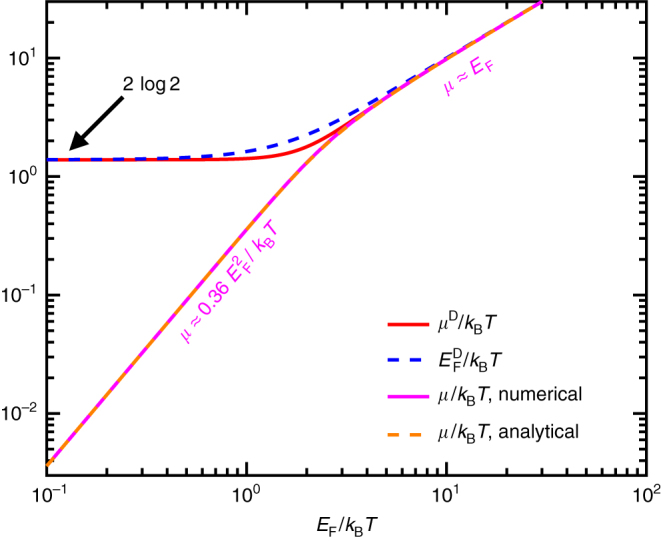
**Graphene chemical potential and Drude weight.** We show the relation between the chemical potential *μ* and the Fermi energy *E*_F_ in graphene, both of them normalized to *k*_B_ *T*. The direct numerical solution of Eq. (20) (*pink solid curve*) is nearly indistinguishable from the analytical expression of Eq. (21) (*dashed orange curve*). For completeness, we also plot the normalized Drude weight *μ* ^D^/*k*_B_ *T* [*red solid curve*, see Eq. (26)] and an approximate Drude weight ${E}_{{\rm{F}}}^{{{\rm D}}}/{k}_{{\rm{B}}}T$ (*dashed blue curve*)

Direct numerical integration of Eq. (20) allows us to obtain *E*_F_/*k*_B_ *T* as a function of *μ*/*k*_B_ *T*. The result is plotted as a pink solid curve in Fig. 6. Additionally, the large and small asymptotic *T* limits of Eq. (20) (see pink labels in Fig. 6) suggest the following approximate relation

21 $${\left(\frac{{E}_{{\rm{F}}}}{{k}_{{\rm{B}}}T}\right)}^{\!\!4}=({\rm{log}}^{2}\,16){\left(\frac{\mu }{{k}_{{\rm{B}}}T}\right)}^{\!\!2}+{\left(\frac{\mu }{{k}_{{\rm{B}}}T}\right)}^{\!\!4},$$

which is in excellent agreement with the full solution of Eq. (20) (cf. pink-solid and dashed-orange curves in Fig. 6). Also note that approximate^113–115^ and asymptotic^116,117^ values for the Drude weight have been proposed to work well in different limits, although they lack the universal accuracy of Eq. (21).

### Electronic heat capacity of graphene

**Fig. 7:**
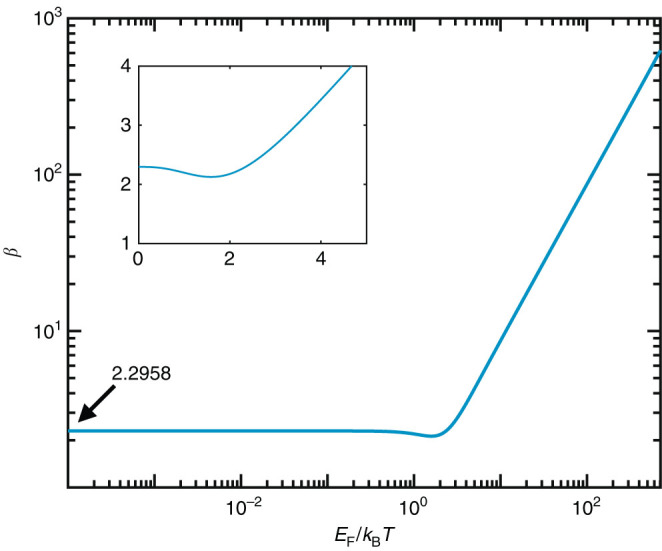
**Graphene electronic heat.** We show the dependence of the thermal coefficient *β* on *E*_F_/*k*_B_ *T*, as calculated from Eq. (24). This parameter permits obtaining the electronic heat per unit of graphene area as $\beta \,{({k}_{{\rm{B}}}T)}^{3}/(\hbar {v}_{{\rm{F}}})^{2}$ [Eq. (23)]. The inset shows *β* in linear scale

The heat capacity is needed to relate the electronic thermal energy *Q* to the electronic temperature *T*. By analogy to Eq. (19), the surface density of electronic thermal energy can be calculated as

22 $$\frac{Q}{A}=\frac{4}{A}\sum _{{{\bf{k}}}_{\parallel }}E\{[{f}_{T}(E)-\theta ({E}_{{\rm{F}}}-E)]-[{f}_{T}(-E)-\theta ({E}_{{\rm{F}}}+E)]\},$$

where the step functions arise when subtracting the energy at *T* = 0 because *f*_*T*=0_(*E*) = *θ*(*E*_F_−*E*). After some straightforward algebra, we find

23 $$\frac{Q}{A}=\beta \,\frac{{({k}_{{\rm{B}}}T)}^{3}}{{(\hbar {v}_{{\rm{F}}})}^{2}},$$

where the thermal coefficient

24 $$\beta =\frac{2}{\pi }\left[{\int }_{\!\!\!\!0}^{\infty }{x}^{2}{\rm d}x\left(\frac{1}{{{\rm{e}}}^{x+\mu /{k}_{{\rm{B}}}T}+1}+\frac{1}{{{\rm{e}}}^{x-\mu /{k}_{{\rm{B}}}T}+1}\right)-\frac{1}{3}{\left(\frac{{E}_{{\rm{F}}}}{{k}_{{\rm{B}}}T}\right)}^{\!\!3}\right]$$

explicitly depends on *μ*/*k*_B_ *T*, which is in turn a function of *E*_F_/*k*_B_ *T* [see Eq. (20)], so we find that *β* is only a function of *E*_F_/*k*_B_ *T*. Numerical evaluation of Eq. (24) yields the results shown in Fig. 7. For *E*_F_ ≪ *k*_B_ *T*, we have $\beta =(4/\pi ){\int }_{0}^{\infty }{\theta }^{2}{\rm d}\theta /(\mathrm{1}+{{\rm{e}}}^{\theta })\approx 2.2958$. (Incidentally, we correct this parameter here for a factor of 2 that was missing in ref. 58.) We note that the graphene heat capacity has been widely used in previous studies^64,114,115^in the so-called degenerate limit (*k*_B_ *T* ≪ *μ*).

### Graphene conductivity

We adopt the local-RPA model for the graphene conductivity^58,83,84^

25 $$\sigma (\omega )=\frac{{e}^{2}}{\pi {\hbar }^{2}}\frac{i}{(\omega +i{\tau }^{-1})}\left\{{\mu }^{{\rm{D}}}-{\int }_{\!\!\!0}^{\infty }{\rm d}E\frac{{f}_{T}(E)-{f}_{T}(-E)}{1-4{E}^{2}/[{\hbar }^{2}{(\omega +i{\tau }^{-1})}^{2}]}\right\},$$

where

26 $${\mu }^{{\rm{D}}}=\mu +2{k}_{{\rm{B}}}T\,\rm{log}(1+{e}^{-\mu /{k}_{{\rm{B}}}T})$$

is a temperature-dependent effective Drude weight that accounts for intraband transitions and has been the object of a recent theoretical and experimental study^90^. The integral term in Eq. (25) represents the contribution from interband transitions. Besides the explicit dependence on temperature *T*, we note that there is an additional dependence through the chemical potential *μ*. We plot the resulting *μ* ^D^ in Fig. 6 (red-solid curve). A reasonable approximation to this parameter is obtained by substituting *E*_F_ for *μ* in Eq. (26) (dashed-blue curve in Fig. 6).

We assume a rather conservative value for the energy broadening $\hbar {\tau }^{-1}\mathrm{=10}\,$ meV throughout this work (this corresponds to a Drude-model mobility^118^ $e{v}_{{\rm{F}}}^{2}\tau /{E}_{{\rm{F}}}\mathrm{=3300}\,$ cm^2^V^−1^s^−1^ for *E*_F_ = 0.2 eV). For simplicity, we further neglect the dependence of *τ* on temperature and chemical potential, which could be readily incorporated following previous studies^114–116^. This dependence is partially absorbed in the assumed value of *τ* over the significant range of temperatures under consideration, although a more detailed analysis could reveal unexpected effects outside that range.

### Time evolution

The temporal evolution of the electronic temperature is given by Eq. (2), which we solve numerically by using a 4th order Runge-Kutta method. It is instructive to rewrite it with the temperatures appearing in a more explicit form. Using the ${Q}_{\ell }$ dependence on ${T}_{\ell }$ given by Eq. (23), we find

$$C({T}_{\ell }){\dot{T}}_{\ell }=-\frac{{T}_{\ell }}{{\tau }_{{\rm{ph}}}}+\frac{4{\hbar }^{2}{v}_{{\rm{F}}}^{2}}{\pi \beta{\rm D}_{\ell }^{2}{k}_{{\rm{B}}}^{3}{T}_{\ell }^{2}}{P}_{\ell }({T}_{1},{T}_{2}),$$

where $C({T}_{\ell })\mathrm{=3}+({T}_{\ell }/\beta )({\rm d}\beta /{\rm d}{T}_{\ell })$ is a dimensionless coefficient that varies between 3 and 4 in the large and small ${T}_{\ell }$ limits, respectively (see *β* dependence on ${T}_{\ell }$ in Fig. 7).

In the simulations of Figs. 2d and 3 and Supplementary Figs. 11 and 12 we fix the initial temperatures ${T}_{\ell }$ to prescribed values. However, in the calculation of Fig. 4 the initial temperatures are determined by the energy absorbed from a light pulse via the absorption cross-section given by Eq. (18). Assuming a *δ*-function pulse of frequency *ω*_0_ and fluence *F*_0_, we have ${Q}_{\ell }(t=0)={\sigma }_{\ell }^{{\rm{abs}}}({\omega }_{0}){F}_{0}$. The initial temperature is then obtained by entering this value of ${Q}_{\ell }$ in Eq. (23).

### Data availability

The data that support the findings of this study are available from the corresponding author upon request.

## Electronic supplementary material

Supplementary Information Supplementary Figures, Supplementary Notes and Supplementary References
