# Supplementary material for: Ultrafast radiative heat transfer
Source: Nat Commun. 2017 Feb 23;8:2. doi: 10.1038/s41467-016-0013-x (PMC5431947; doi:10.1038/s41467-016-0013-x)
Supplement: Supplementary file 1 — Supplementary Figures, Supplementary Notes and Supplementary References [file 41467_2016_13_MOESM1_ESM.pdf]

Supplementary Note 1: Derivation of Eq. (6) of the main paper

We provide a brief derivation of the fluctuation-dissipation theorem[1,2] (FDT) for fluctuations of the charge density in the frequency domain. We start by considering a system characterized by its charge density operator $\rho(\mathbf{r},t)$ in the Heisenberg picture and described through the Hamiltonian $H = H_0 + H_1$, where H_0 is the unperturbed term, while

$$H_1 = \int d^3\mathbf{r}\, \rho(\mathbf{r},t)\phi(\mathbf{r},t) \tag{1}$$

accounts for the time-dependent interaction between ρ and an external electric potential $\phi(\mathbf{r},t)$. Using first-order perturbation theory under the assumption that H_1 vanishes in the $t \to -\infty$ limit, we can write the eigenstates of the perturbed system as

$$|\psi_m(t)\rangle \approx |m\rangle - \frac{i}{\hbar}\int_{-\infty}^{t} dt'\, H_1(t')\, |m\rangle\,, \tag{2}$$

where $|m\rangle$ is the eigenstate of H_0 with energy E_m (i.e., $H_0\,|m\rangle = E_m\,|m\rangle$). Summing the contributions from all perturbed states $|\psi_m(t)\rangle$, we obtain the expectation value of the charge density induced by H_1 as

$$\rho^{\rm ind}(\mathbf{r},t) = \langle \rho(\mathbf{r},t) - \rho(\mathbf{r},-\infty)\rangle = \int dt' \int d^3\mathbf{r}'\; \chi(\mathbf{r},\mathbf{r}',t-t')\phi(\mathbf{r}',t'),$$

where

$$\chi(\mathbf{r},\mathbf{r}',t-t') = -\frac{i}{\hbar}\theta(t-t')\sum_m \frac{{\rm e}^{-E_m/k_BT}}{Z}\,\langle m|\,[\rho(\mathbf{r},t),\rho(\mathbf{r}',t')]\,|m\rangle \tag{3}$$

is the electric susceptibility of the system and $Z = \sum_m {\rm e}^{-E_m/k_BT}$ is the partition function at temperature T. The former can be expressed in the frequency domain by considering a harmonic potential ϕ with a ${\rm e}^{-i\omega t}$ time dependence that is directly transmitted to the induced charge. We find

$$\chi(\mathbf{r},\mathbf{r}',\omega) = \int dt'\; \chi(\mathbf{r},\mathbf{r}',t-t'){\rm e}^{-i\omega(t'-t)} = \frac{1}{Z}\sum_{m,n}\langle m|\,\rho(\mathbf{r})\,|n\rangle\,\langle n|\,\rho(\mathbf{r}')\,|m\rangle\,\frac{{\rm e}^{-E_m/k_BT} - {\rm e}^{-E_n/k_BT}}{\hbar\omega + E_m - E_n + i0^+}, \tag{4}$$

where we have used $\rho(\mathbf{r},t) = {\rm e}^{iH_0t/\hbar}\rho(\mathbf{r}){\rm e}^{-iH_0t/\hbar}$ as well as the closure relation $\sum_n |n\rangle\,\langle n| = \mathbb{I}$.

At this point, we follow a similar procedure for calculating the self correlations of the fluctuating charge density $\rho^{\rm fl}$. We obtain

$$\begin{aligned}\langle \rho^{\rm fl}(\mathbf{r},\omega)\rho^{\rm fl}(\mathbf{r}',\omega')\rangle &= \int dt \int dt'\; {\rm e}^{i\omega t}{\rm e}^{i\omega' t'}\;\langle \rho^{\rm fl}(\mathbf{r},t)\rho^{\rm fl}(\mathbf{r}',t')\rangle \\ &= \frac{1}{Z}\int dt \int dt'\; {\rm e}^{i\omega t}{\rm e}^{i\omega' t'}\sum_{m,n}{\rm e}^{-E_m/k_BT}{\rm e}^{i(E_m-E_n)(t-t')/\hbar}\,\langle m|\,\rho(\mathbf{r})\,|n\rangle\,\langle n|\,\rho(\mathbf{r}')\,|m\rangle \\ &= 2\pi\delta(\omega+\omega')S(\omega),\end{aligned} \tag{5}$$

where

$$S(\omega) = \frac{2\pi\hbar}{Z}\sum_{m,n}{\rm e}^{-E_m/k_BT}\,\langle m|\,\rho(\mathbf{r})\,|n\rangle\,\langle n|\,\rho(\mathbf{r}')\,|m\rangle\,\delta(\hbar\omega + E_m - E_n). \tag{6}$$

Comparing this expression with Eq. (4), we obtain $S(\omega) = -2\hbar\,[n(\omega)+1]\,{\rm Im}\{\chi(\mathbf{r},\mathbf{r}',\omega)\}$, where $n(\omega) = [{\rm e}^{\hbar\omega/k_BT}-1]^{-1}$ is the Bose-Einstein distribution function. We conclude that

$$\langle \rho^{\rm fl}(\mathbf{r},\omega)\rho^{\rm fl}(\mathbf{r}',\omega')\rangle = -4\pi\hbar\delta(\omega+\omega')\;[n(\omega)+1]\;{\rm Im}\{\chi(\mathbf{r},\mathbf{r}',\omega)\}. \tag{7}$$

Additionally, interchanging $\rho(\mathbf{r},\omega)$ and $\rho(\mathbf{r}',\omega')$, we find

$$\langle \rho^{\rm fl}(\mathbf{r}',\omega')\rho^{\rm fl}(\mathbf{r},\omega)\rangle = -4\pi\hbar\delta(\omega+\omega')\;n(\omega)\;{\rm Im}\{\chi(\mathbf{r},\mathbf{r}',\omega)\}, \tag{8}$$

where we have used the properties $\chi(\mathbf{r},\mathbf{r}',\omega) = \chi(\mathbf{r}',\mathbf{r},\omega)$, $\chi(\mathbf{r},\mathbf{r}',-\omega) = \chi^*(\mathbf{r},\mathbf{r}',\omega)$, and $n(-\omega)+1 = -n(\omega)$. Finally, the expectation value of the physically meaningful symmetrized correlation becomes

$$\langle \rho^{\rm fl}(\mathbf{r}',\omega')\rho^{\rm fl}(\mathbf{r},\omega)\rangle_{\rm sym} = \frac{1}{2}\left[\langle \rho^{\rm fl}(\mathbf{r},\omega)\rho^{\rm fl}(\mathbf{r}',\omega')\rangle + \langle \rho^{\rm fl}(\mathbf{r}',\omega')\rho^{\rm fl}(\mathbf{r},\omega)\rangle\right] = -4\pi\hbar\delta(\omega+\omega')\;[n(\omega)+\frac{1}{2}]\;{\rm Im}\{\chi(\mathbf{r},\mathbf{r}',\omega)\}. \tag{9}$$

This is the FDT used in the main paper [see Eq. (6) there], where we drop the 'sym' subscript for clarity.

Supplementary Note 2: Derivation of Eqs. (7) and (8) of the main paper

We start from Eq. (4) of the main paper, which we recast as

$$P_{1\leftarrow 2} = i \iint \frac{d\omega d\omega'}{(2\pi)^2}\, \omega\, \mathrm{e}^{-i(\omega+\omega')t} \int d^3\mathbf{r} \int d^3\mathbf{r}' \left\langle \left[\left(\rho_2^{\rm fl}(\omega)\right)^{\rm T} \cdot \Delta^{\rm T}(\omega) \cdot v \cdot \chi_1(\omega) \cdot v \cdot \Delta(\omega') \cdot \rho_2^{\rm fl}(\omega') \right] \Big|_{\mathbf{r},\mathbf{r}'} \right\rangle . \quad (10)$$

This expression can be conveniently rewritten by moving $\left(\rho_2^{\rm fl}(\omega)\right)^{\rm T}$ to the right end as

$$P_{1\leftarrow 2} = i \iint \frac{d\omega d\omega'}{(2\pi)^2}\, \omega\, \mathrm{e}^{-i(\omega+\omega')t} \int d^3\mathbf{r} \int d^3\mathbf{r}' \left[\Delta^{\rm T}(\omega) \cdot v \cdot \chi_1(\omega) \cdot v \cdot \Delta(\omega') \cdot \left\langle \rho_2^{\rm fl}(\omega') \cdot \left(\rho_2^{\rm fl}(\omega)\right)^{\rm T} \right\rangle \right] \Big|_{\mathbf{r},\mathbf{r}'} . \quad (11)$$

Here, $\rho_2^{\rm fl}(\omega') \cdot \left(\rho_2^{\rm fl}(\omega)\right)^{\rm T}$ is a matrix formed by the product of column and row vectors. Charge fluctuations are readily evaluated using the FTD [Eq. (6) of the main paper] together with the identity $\chi_\ell(\omega) = \chi_\ell^*(-\omega)$. We find

$$\begin{aligned} P_{1\leftarrow 2} &= \frac{-i\hbar}{\pi} \int \omega\, d\omega\, (n_2 + 1/2)\ \mathrm{Tr}\left[\Delta^{\rm T} \cdot v \cdot \chi_1 \cdot v \cdot \Delta^* \cdot \mathrm{Im}\{\chi_2\} \right] \\ &= \frac{-i\hbar}{\pi} \int \omega\, d\omega\, (n_2 + 1/2)\ \mathrm{Tr}\left[\mathrm{Im}\{\chi_2\} \cdot \Delta^\dagger \cdot v \cdot \chi_1 \cdot v \cdot \Delta \right], \end{aligned} \quad (12)$$

where the second line is obtained from the first one by applying the matrix trace identity $\mathrm{Tr}[A] = \mathrm{Tr}[A^{\rm T}]$ as well as $v = v^{\rm T}$ and $\chi_\ell = \chi_\ell^{\rm T}$. We note that a dependence of χ_ℓ, Δ, and n_2 on ω is understood. We now split the integral as $\int d\omega \to \int_0^\infty d\omega + \int_{-\infty}^0 d\omega$ and change ω to $-\omega$ in the negative frequency term. Using the property $[n_\ell(\omega) + 1/2] = -[n_\ell(-\omega) + 1/2]$, we obtain

$$P_{1\leftarrow 2} = \frac{-i\hbar}{\pi} \int_0^\infty \omega\, d\omega\, (n_2 + 1/2)\ \mathrm{Tr}\left[\mathrm{Im}\{\chi_2\} \cdot \Delta^\dagger \cdot v \cdot \chi_1 \cdot v \cdot \Delta - \mathrm{Im}\{\chi_2\} \cdot \Delta^{\rm T} \cdot v \cdot \chi_1^* \cdot v \cdot \Delta^* \right]. \quad (13)$$

Taking the transpose of the second term and using the above matrix properties together with $\mathrm{Tr}[A \cdot B] = \mathrm{Tr}[B \cdot A]$, Eq. (13) reduces to

$$\begin{aligned} P_{1\leftarrow 2} &= \frac{-i\hbar}{\pi} \int_0^\infty \omega\, d\omega\, (n_2 + 1/2)\ \mathrm{Tr}\left[\mathrm{Im}\{\chi_2\} \cdot \Delta^\dagger \cdot v \cdot \chi_1 \cdot v \cdot \Delta - \Delta^\dagger \cdot v \cdot \chi_1^* \cdot v \cdot \Delta \cdot \mathrm{Im}\{\chi_2\} \right] \\ &= \frac{-i\hbar}{\pi} \int_0^\infty \omega\, d\omega\, (n_2 + 1/2)\ \mathrm{Tr}\left[\Delta^\dagger \cdot v \cdot \chi_1 \cdot v \cdot \Delta \cdot \mathrm{Im}\{\chi_2\} - \Delta^\dagger \cdot v \cdot \chi_1^* \cdot v \cdot \Delta \cdot \mathrm{Im}\{\chi_2\} \right] \\ &= \frac{-i\hbar}{\pi} \int_0^\infty \omega\, d\omega\, (n_2 + 1/2)\ \mathrm{Tr}\left[\Delta^\dagger \cdot v \cdot (\chi_1 - \chi_1^*) \cdot v \cdot \Delta \cdot \mathrm{Im}\{\chi_2\} \right] \\ &= \frac{2\hbar}{\pi} \int_0^\infty \omega\, d\omega\, (n_2 + 1/2)\ \mathrm{Tr}\left[\Delta^\dagger \cdot v \cdot \mathrm{Im}\{\chi_1\} \cdot v \cdot \Delta \cdot \mathrm{Im}\{\chi_2\} \right], \end{aligned} \quad (14)$$

which is Eq. (7) of the main paper. A similar argument can be followed to prove that $P_{1\leftarrow 2}$ is indeed a real number.

When interchanging the subindices $1 \Leftrightarrow 2$, upon inspection of the definition of Δ [Eq. (5) of the main paper], we have $v \cdot \Delta \Leftrightarrow \Delta^{\rm T} \cdot v$. Using this transformation, as well as the trace properties noted above, we find that the expression in the square brackets of Eq. (7) of the main paper remains the same upon index interchange. This directly leads to Eq. (8) of the main paper for the difference $P_{2\leftarrow 1} - P_{1\leftarrow 2}$.

Supplementary Note 3: Computation of $v_{jj'}$ for coaxial disks

In this section, we provide a computationally efficient expression to calculate the Coulomb interaction matrix elements $v_{jj'}$ [Eq. (14) of the main paper] for coaxial disks [i.e., with the plasmon wave functions (PWFs) of Eqs. (16) of the main paper]. We start by rewriting the Coulomb potential as[3]

$$\frac{1}{|\mathbf{r} - \mathbf{r}'|} = 4\pi \sum_{l=0}^{\infty} \sum_{m=-l}^{l} \frac{1}{2l+1} \frac{r_<^l}{r_>^{l+1}} Y_{lm}(\Omega_{\mathbf{r}}) Y_{lm}^*(\Omega_{\mathbf{r}'}), \quad (15)$$

where Y_L are spherical harmonics, $r_< = \min\{r, r'\}$, and $r_> = \max\{r, r'\}$. Specifying Eq. (14) of the main paper for two PWFs $\rho^{\kappa}_{m\nu}(\vec{\theta})$ and $\rho^{\kappa'}_{m'\nu'}(\vec{\theta}')$, where κ and κ' are either c or s for cos and sin azimuthal dependences, respectively, and using Eq. (15), we can perform the azimuthal integrals of $\vec{\theta}$ and $\vec{\theta}'$ analytically by choosing the spatial origin at a point along the axis of revolution symmetry in between the two disks. Upon detailed examination, we find $v_{jj'}$ to be zero unless $m = m'$ and $\kappa = \kappa'$. Therefore, PWFs of different azimuthal symmetry do not interact. It should be also noted that only $\kappa = \mathrm{c}$ contributes to $m = 0$. The remaining nonzero elements are independent of κ, but they depend on m, ν, and ν' as

$$v^m_{\nu\nu'} = (1+\delta_{m0})\,\frac{8\pi^3 D_1^2 D_2^2}{\epsilon}\sum_{l=m}^{\infty}\frac{1}{2l+1}\int_0^{1/2}\theta\,d\theta\,\rho_{m\nu}(\theta)\int_0^{1/2}\theta' d\theta'\,\rho_{m\nu'}(\theta')\,\frac{r_<^l}{r_>^{l+1}}\,Y_{lm}(\theta_1,0)\,Y_{lm}(\theta_2,0), \tag{16}$$

where we take $r = D_1\theta$ and $r' = D_2\theta'$. Additionally, the spherical harmonics in this expression are evaluated at zero azimuthal angle, while the polar angles are $\theta_1 = \tan^{-1}(D_1\theta/d_1)$ and $\theta_2 = \pi - \tan^{-1}(D_2\theta'/d_2)$, where d_1 and d_2 are the distances from the disks to the origin (i.e., $d_1 + d_2 = d$), a convenient choice being $d_1 = d$ and $d_2 = 0$, so that $(r_</r_>)^l$ goes rapidly down for large l, particularly at large separations. Equation (16) gives the $(\nu\nu')$ elements of the matrix v^m entering Eq. (1) of the main paper. This expression is also useful to normalize the PWFs via Eq. (13) of the main paper, whose integral corresponds to $v^m_{\nu\nu'}$ with $\epsilon = 1$, $D_1 = D_2$, and $d = 0$.

Supplementary Note 4: Radiative heat transfer between extended graphene films

The lack of translational invariance in graphene disks prevents us from including nonlocal effect in the classical description of their optical response. In order to assess the relative contribution of such effects, we consider extended graphene films, for which the nonlocal conductivity admits analytical expressions[4,5]. The radiative heat transfer power can then be decomposed in components associated with different parallel wave vectors $\mathbf{k}_\parallel$. We argue that the relative importance of nonlocal contributions for a disk of diameter D is roughly the same as for the $k_\parallel = 2\pi/D$ component in the extended films. An expression for the transfer power between films can be obtained by starting from Eq. (8) of the main paper, replacing the trace by the sum $\sum_{\mathbf{k}_\parallel} \rightarrow (A/4\pi^2)\int d^2\mathbf{k}_\parallel$, where A is the film area, and writing $v \rightarrow (2\pi/k_\parallel)\mathrm{e}^{-k_\parallel d}$ for the Coulomb interaction in $\mathbf{k}_\parallel$ space. Additionally, from a direct analysis of the electrostatic problem, we have $v\cdot\chi_\ell \rightarrow -r_\ell$, where $r_\ell = 1/(1 - i\omega/2\pi k_\parallel\sigma_\ell)$ is the graphene reflection coefficient for TM polarization (notice that the reflection for TE polarization vanishes in the quasistatic limit), while σ_ℓ is the conductivity of the layer $\ell = 1, 2$. Putting these elements together, the transfer power per unit area becomes $\int_0^\infty dk_\parallel P(k_\parallel)/A$, where

$$\frac{1}{A}P(k_\parallel) = \frac{\hbar k_\parallel}{\pi^2}\int_0^\infty \omega d\omega (n_1 - n_2)\mathrm{e}^{-2k_\parallel d}\frac{\mathrm{Im}\{r_1\}\mathrm{Im}\{r_2\}}{\left|1 - r_1 r_2 \exp(-2k_\parallel d)\right|^2}, \tag{17}$$

in agreement with the $c \rightarrow \infty$ limit of the well-known expression for the transfer power between two planar structures[6]. We plot this quantity in Supplementary Fig. 4 using the full nonlocal RPA (broken curves) and the local-RPA (solid curves) models for the conductivity. The agreement between these results indicates that nonlocal effects only play a marginal role in this study.

Supplementary References

1 Nyquist, H. Thermal agitation of electric charge in conductors. *Phys. Rev.* **32**, 110–113 (1928).
2 Callen, H. B. & Welton, T. A. Irreversibility and generalized noise. *Phys. Rev.* **83**, 34–40 (1951).
3 Jackson, J. D. *Classical Electrodynamics* (Wiley, 1999).
4 Wunsch, B., Stauber, T., Sols, F. & Guinea, F. Dynamical polarization of graphene at finite doping. *New J. Phys.* **8**, 318 (2006).
5 Hwang, E. H. & Das Sarma, S. Dielectric function, screening, and plasmons in two-dimensional graphene. *Phys. Rev. B* **75**, 205418 (2007).
6 Svetovoy, V. B., van Zwol, P. J. & Chevrier, J. Plasmon enhanced near-field radiative heat transfer for graphene covered dielectrics. *Phys. Rev. B* **85**, 155418 (2012).
7 Thongrattanasiri, S., Manjavacas, A. & García de Abajo, F. J. Quantum finite-size effects in graphene plasmons. *ACS Nano* **6**, 1766–1775 (2012).

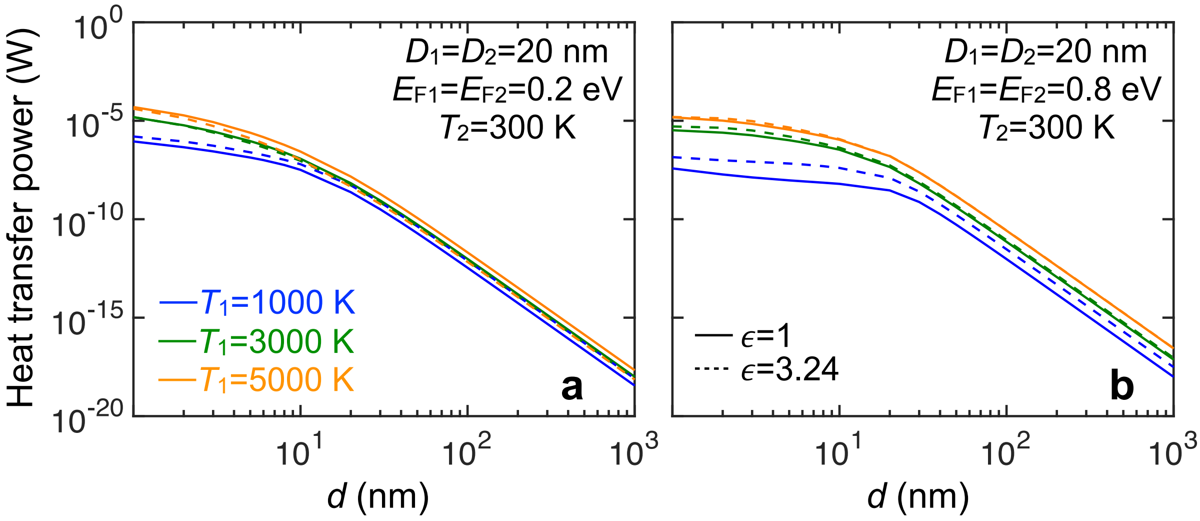

Supplementary Figure 1: **Influence of dielectric environment on the radiative heat transfer power (HTP).** We show the dependence of the HTP on the separation distance d between two graphene nanodisks (20 nm diameter) for different values of T_1 (see legend) and fixed $T_2 = 300\,$K. The disks are doped to a Fermi energy $E_{\rm F} = 0.2\,$eV in (a) and 0.8 eV in (b). We consider homogeneous media of permittivity $\epsilon = 1$ (vacuum, solid curves) or $\epsilon = 3.24$ (broken curves) at the thermal wavelengths under consideration. The graphene is described using the local-RPA conductivity. A damping energy $\hbar\tau^{-1} = 10\,$meV is assumed in all figures, unless otherwise stated.

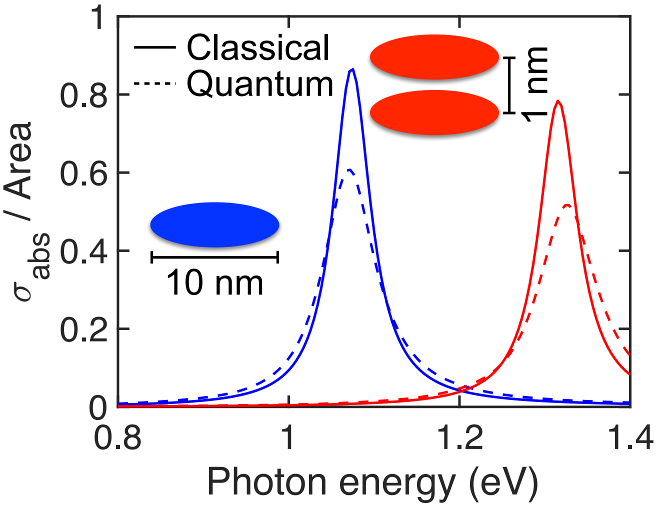

Supplementary Figure 2: **Influence of nonlocal effects in the response of graphene: absorption cross-section.** Absorption cross-section of individual (blue) and closely spaced (red) graphene disks calculated using either classical (local-RPA conductivity, solid curves) or quantum-mechanical (tight-binding combined with full RPA, as described elsewhere[7], broken curves) models. We assume a Fermi energy of 2 eV and a damping of 0.05 eV.

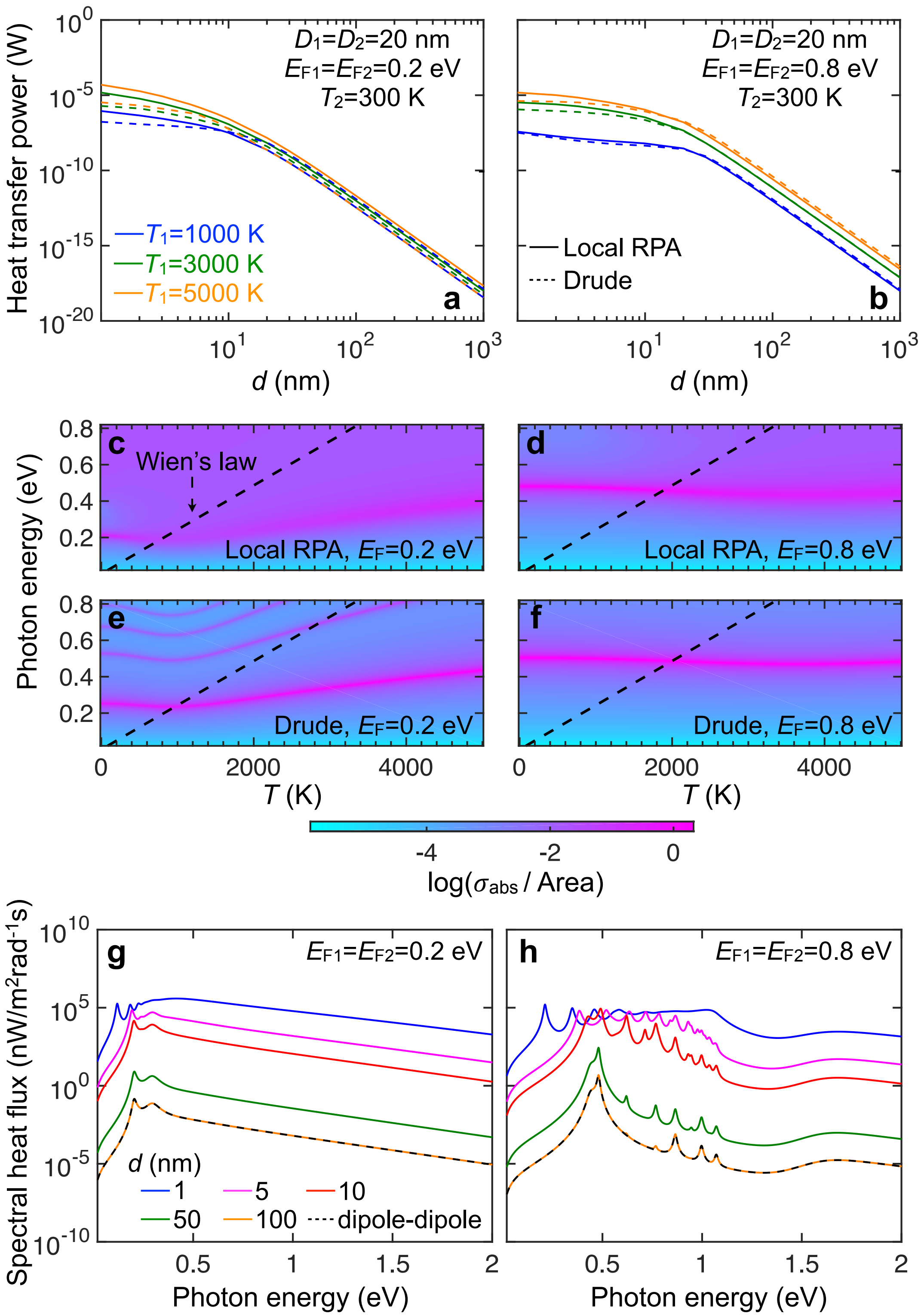

Supplementary Figure 3: **Dependence of the HTP on the model used for the graphene conductivity.** **(a)** Dependence of the HTP on the separation distance d between two graphene nanodisks (20 nm diameter, 0.2 eV Fermi energy) plotted for different values of T_1 (see legend) and fixed $T_2 = 300\,\mathrm{K}$. Solid (dashed) curves are obtained with the local-RPA (Drude) model for the graphene conductivity. The temperature-dependent local-RPA is given by Eq. (25) of the main paper, while the Drude model is obtained by neglecting the integral term in that equation. **(b)** Same as (a) for 0.8 eV Fermi energy. **(c-f)** Absorption cross-section of a 20 nm graphene disk as a function of photon energy $\hbar\omega$ and temperature T calculated for different values of the Fermi energy using the two models considered for the conductivity (see labels). The temperature dependence enters through the conductivity [see Eq. (25) of the main paper]. The dashed lines correspond to Wien's law, $\hbar\omega \approx 2.82\,k_{\rm B}T$. **(g,h)** Spectral dependence of the HTP for 20 nm graphene disks. The vertical axis shows the value of the integrand in Eq. (8) of the main paper. The hot (cold) disk is at temperature $T_1 = 3000\,\mathrm{K}$ ($T_2 = 300\,\mathrm{K}$). We consider different values of the disk separation d and Fermi energies (see labels). The dipole-dipole approximation is shown for $d = 100\,\mathrm{nm}$ (dashed curves).

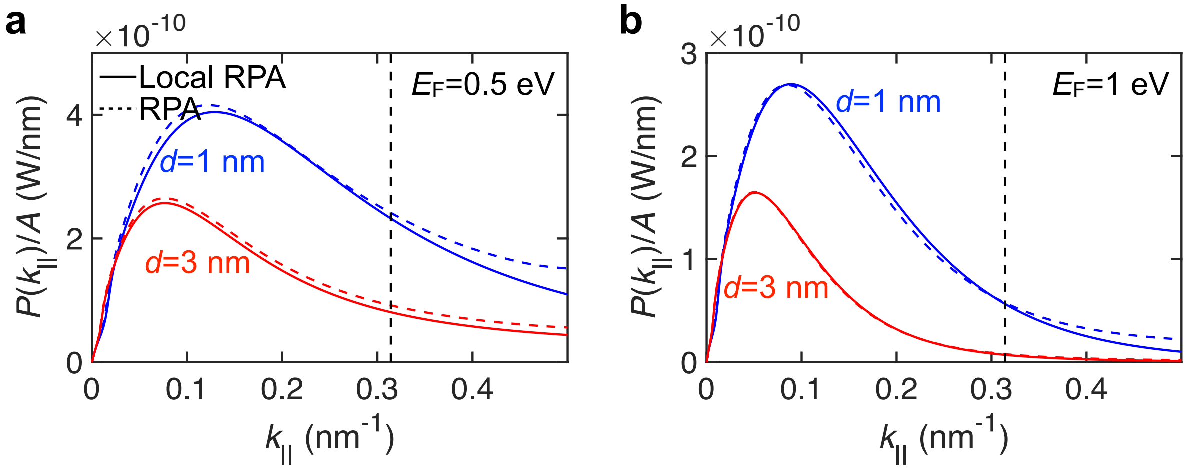

Supplementary Figure 4: **Influence of nonlocal effects in the response of graphene: radiative heat transfer.** We show the heat transfer power per unit area $P(k_\parallel)/A$ between two closely spaced extended graphene films resolved in parallel wave-vector components for combinations of two different Fermi energies and separations (see labels). We assume temperatures $T_1 = 1000\,\mathrm{K}$ and $T_2 = 300\,\mathrm{K}$ in the layers. The conductivity of graphene is described in the full RPA (broken curves) and in the local-RPA limit (solid curves) for a damping of 0.01 eV. The temperature-depencence of the conductivity is neglected for simplicity. The vertical dashed lines at $k_\parallel = 2\pi/D$ qualitatively indicate the region where a maximum contribution is expected for disks of diameter $D = 20\,\mathrm{nm}$.

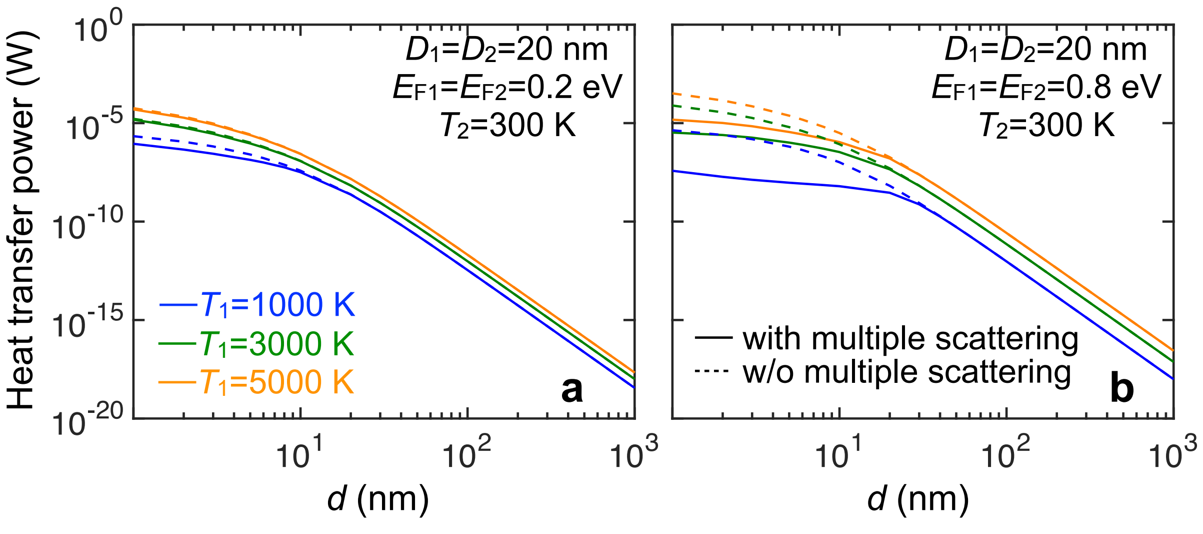

Supplementary Figure 5: **Influence of multiple scattering in the optical interaction between graphene disks on the HTP.** **(a)** HTP under the same conditions as in Supplementary Fig. 3(a) (local-RPA) calculated with [solid curves, Eq. (1) of the main paper] and without [dashed curves, same equation with $\Delta^m = \mathbb{I}$] inclusion of multiple scattering in the interaction between the disks. **(b)** Same as (a) for 0.8 eV Fermi energy.

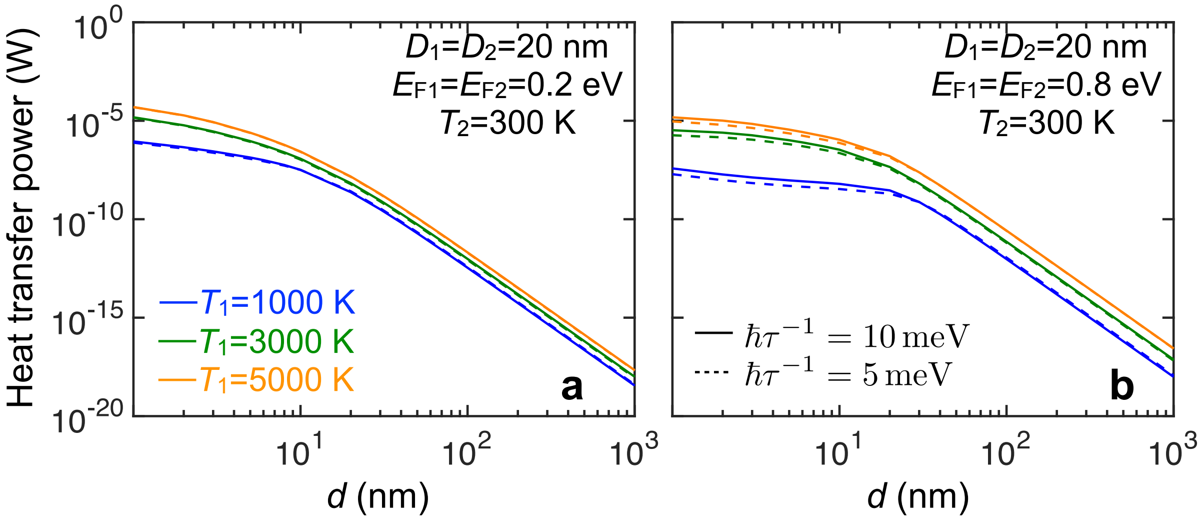

Supplementary Figure 6: **Influence of electronic damping on the HTP.** We show the HTP under the same conditions as in Supplementary Fig. 3(a) (local-RPA) for two different values of the inelastic broadening $\hbar\tau^{-1}$ (see labels). All other plots in this work are obtained with $\hbar\tau^{-1} = 10\,\mathrm{meV}$.

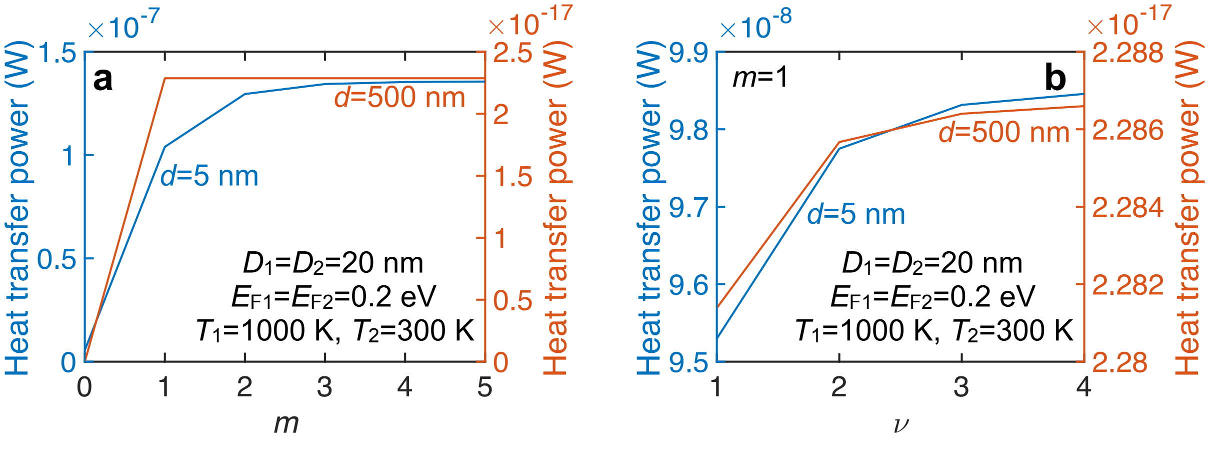

Supplementary Figure 7: **Convergence of the HTP with the number of PWFs.** **(a)** Convergence of the HTP with increasing number of m's included in the calculation under the same conditions as in Supplementary Fig. 3(a) (local-RPA) for two different disk separations d (see labels). **(b)** Convergence of the $m = 1$ contribution to (a) as a function of the number of PWFs included in the calculation.

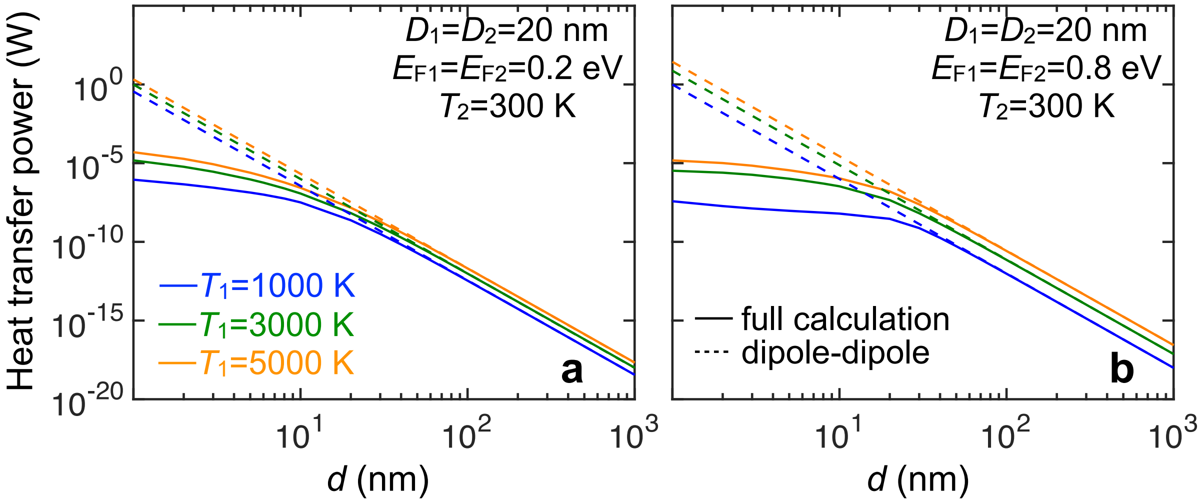

Supplementary Figure 8: **Influence of multipolar orders on the HTP.** We show the HTP under the same conditions as in Supplementary Fig. 3(a) (local-RPA) calculated with the full formalism [solid curves, Eq. (1) of the main paper] and in the dipole-dipole approximation [dashed curves, Eq. (9) of the main paper].

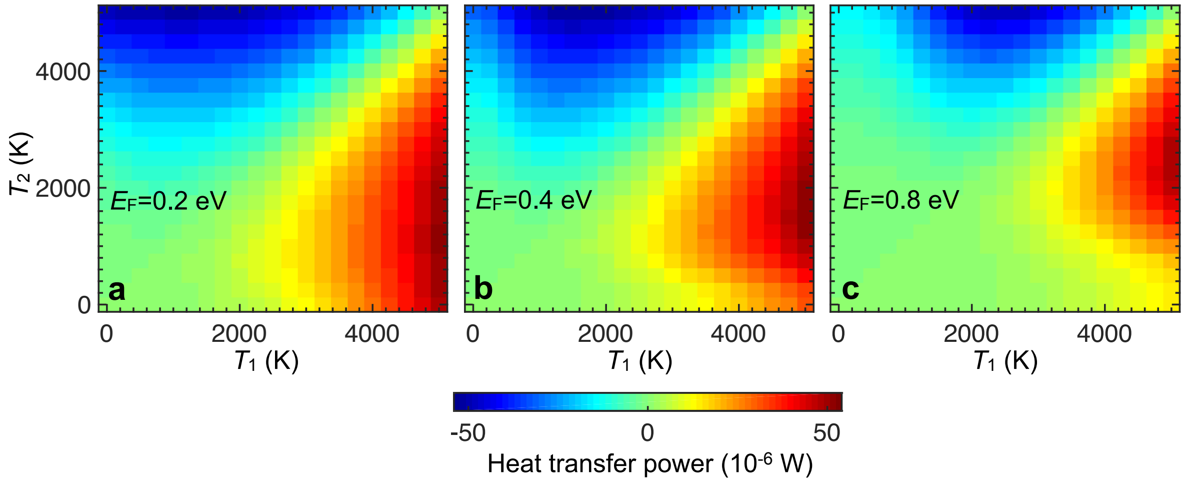

Supplementary Figure 9: **Temperature dependence of the HTP for different doping levels.** We consider two graphene disks of 20 nm diameter separated by a distance $d = 1$ nm. Both disks are doped with the same Fermi energy (see labels).

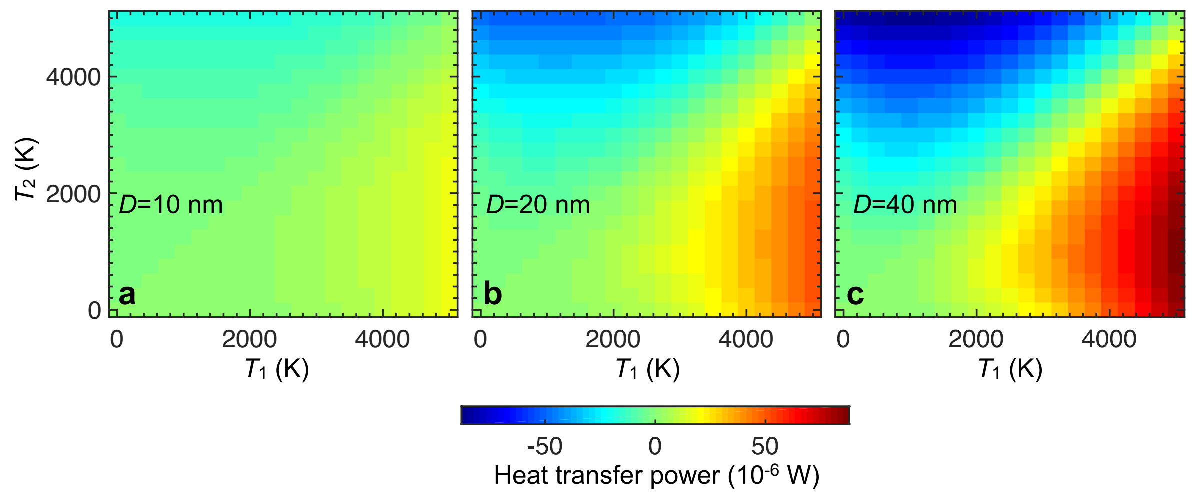

Supplementary Figure 10: **Temperature dependence of the HTP for different disk sizes.** Same as Supplementary Fig. 9 for fixed Fermi energy $E_{F1} = E_{F2} = 0.2\,\text{eV}$ and identical disks for three different values of the diameters (see labels).

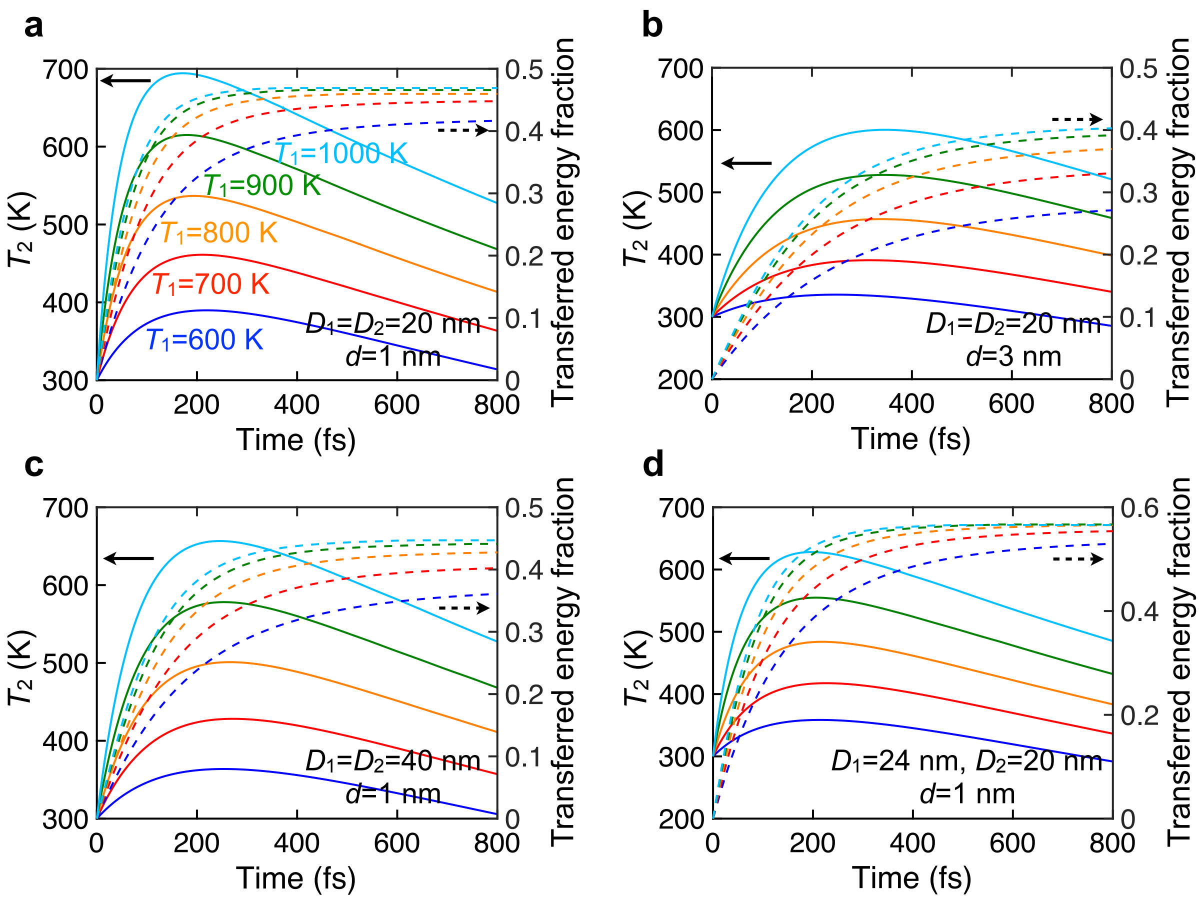

Supplementary Figure 11: **Size and separation dependence of the heat transfer dynamics. I.** Same as Fig. 3(b) of the main paper for different combinations of the disk diameters D_ℓ and the separation d: **(a)** $D_1 = D_2 = 20\,\text{nm}$, $d = 1\,\text{nm}$ [same as Fig. 3(b)]; **(b)** $D_1 = D_2 = 20\,\text{nm}$, $d = 3\,\text{nm}$ (larger spacing); **(c)** $D_1 = D_2 = 40\,\text{nm}$, $d = 1\,\text{nm}$ (larger disks); **(d)** $D_1 = 24\,\text{nm}$, $D_2 = 20\,\text{nm}$, $d = 1\,\text{nm}$ (dissimilar disks). The doping level is $E_F = 0.2\,\text{eV}$ in all cases.

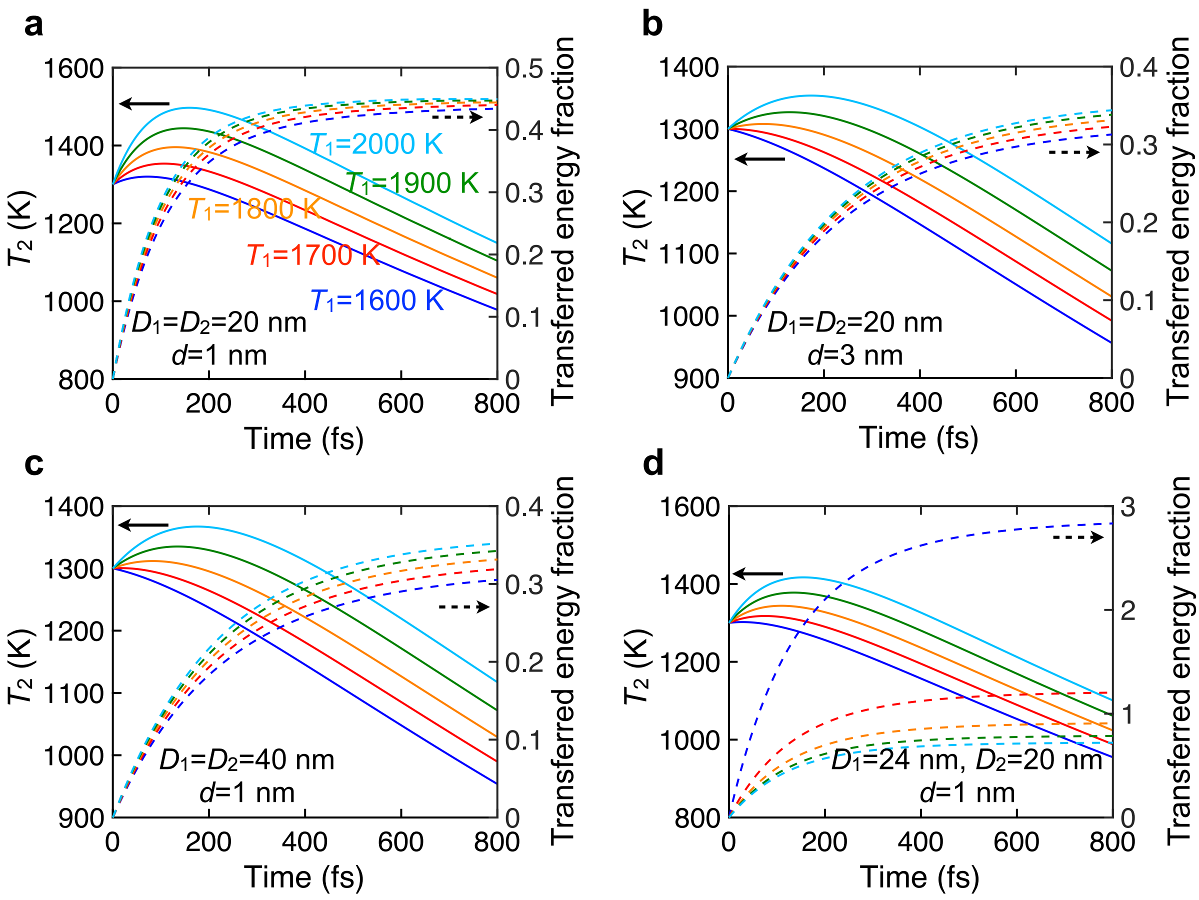

Supplementary Figure 12: **Size and separation dependence of the heat transfer dynamics. II.** Same as Supplementary Fig. 11 for $E_\mathrm{F} = 0.5\,\mathrm{eV}$ and different initial temperatures.

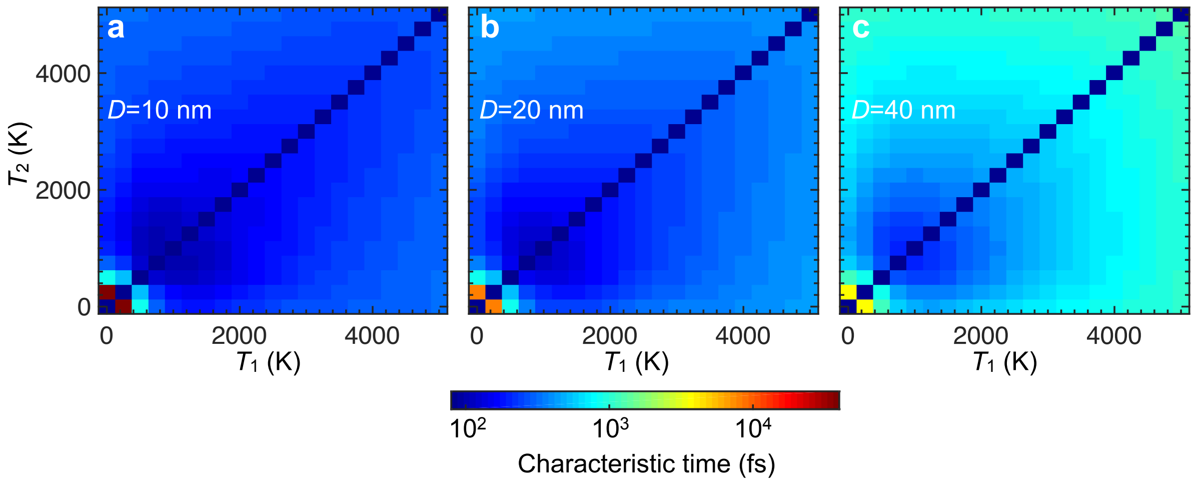

Supplementary Figure 13: **Temperature and size dependence of the characteristic time associated with radiative heat transfer.** We plot the heat transfer time under the same conditions as in Supplementary Fig. 10.
